# Interpretable Multimodal Fusion Model for Bridged Histology and Genomics Survival Prediction in Pan‐Cancer

**DOI:** 10.1002/advs.202407060

**Published:** 2025-03-07

**Authors:** Feng Gao, Junxiang Ding, Baowen Gai, Du Cai, Chuling Hu, Feng‐Ao Wang, Ruikun He, Junwei Liu, Yixue Li, Xiao‐Jian Wu

**Affiliations:** ^1^ Department of General Surgery (Department of Colorectal Surgery) The Sixth Affiliated Hospital Sun Yat‐sen University Guangzhou 510655 China; ^2^ Guangdong Provincial Key Laboratory of Colorectal and Pelvic Floor Diseases The Sixth Affiliated Hospital Sun Yat‐sen University Guangzhou 510655 China; ^3^ Biomedical Innovation Center The Sixth Affiliated Hospital Sun Yat‐sen University Guangzhou 510655 China; ^4^ Shanghai Artificial Intelligence Laboratory Shanghai 200031 China; ^5^ Guangzhou National Laboratory Guangzhou 510005 China; ^6^ Key Laboratory of Systems Health Science of Zhejiang Province School of Life Science Hangzhou Institute for Advanced Study University of Chinese Academy of Sciences Hangzhou 310024 China; ^7^ BYHEALTH Institute of Nutrition & Health Guangzhou 510000 China; ^8^ GZMU‐GIBH Joint School of Life Sciences The Guangdong‐Hong Kong‐Macau Joint Laboratory for Cell Fate Regulation and Diseases Guangzhou Medical University Guangzhou 511436 China; ^9^ School of Life Sciences and Biotechnology Shanghai Jiao Tong University Shanghai 200240 China; ^10^ Shanghai Institute of Nutrition and Health Chinese Academy of Sciences Shanghai 200030 China; ^11^ Collaborative Innovation Center for Genetics and Development Fudan University Shanghai 200433 China; ^12^ Shanghai Institute for Biomedical and Pharmaceutical Technologies Shanghai 200032 China

**Keywords:** artificial intelligence, interpretable bridged multimodal fusion model, pan‐cancer, survival prediction

## Abstract

Understanding the prognosis of cancer patients is crucial for enabling precise diagnosis and treatment by clinical practitioners. Multimodal fusion models based on artificial intelligence (AI) offer a comprehensive depiction of the tumor heterogeneity landscape, facilitating more accurate predictions of cancer patient prognosis. However, in the real‐world, the lack of complete multimodal data from patients often hinders the practical clinical utility of such models. To address this limitation, an interpretable bridged multimodal fusion model is developed that combines histopathology, genomics, and transcriptomics. This model assists clinical practitioners in achieving more precise prognosis predictions, particularly when patients lack corresponding molecular features. The predictive capabilities of the model are validated across 12 cancer types, achieving optimal performance in both complete and missing modalities. The work highlights the promise of developing a clinically applicable medical multimodal fusion model. This not only aids in reducing the healthcare burden on cancer patients but also provides improved assistance for clinical practitioners in precise diagnosis and treatment.

## Graphical abstract

This article introduces the Brim model, an interpretable multimodal fusion framework that integrates histopathology, genomics, and transcriptomics to enhance cancer prognosis prediction. Addressing real‐world data limitations, the model enables precise predictions even with incomplete molecular data. Validated across 12 cancer types, Brim demonstrates superior performance and clinical applicability, supporting individualized treatment decisions while reducing healthcare burdens for patients.

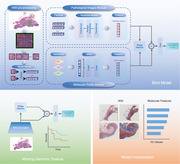

## 1 Introduction

Cancer, due to its complex pathogenic mechanisms and a high degree of heterogeneity, often leads to low treatment response rates and unpredictable patient outcomes.^[^ ^1^ ^]^ The rapid development of artificial intelligence (AI) technology has driven its deep application in cancer research and oncology, including cancer detection and diagnosis, subtype classification, and the identification of new therapeutic targets.^[^ ^2^, ^3^ ^]^ Given the success of AI in visual tasks, their applications in cancer mostly focus on using images to characterize cancers, such as automatically and accurately detecting different cancer phenotypes from pathological or radiological images. Utilizing deep learning methods to analyze pathological whole‐slide images (WSIs) helps doctors capture tumor microenvironment information that is beyond the reach of the human eye, significantly improving the accuracy of cancer diagnosis and prognosis prediction.^[^ ^4^, ^5^, ^6^ ^]^ Due to the substantial size and ultra‐high resolution of whole slide images (WSIs) in pathology, deep learning techniques often necessitate segmenting these WSIs into smaller patches to enable efficient high‐performance computing. For example, Deep Sets proposes the use of sum pooling to aggregate patch‐level features into slide‐level features. Attention MIL builds on Deep Sets by replacing sum pooling with global attention pooling.^[^ ^7^ ^]^ DeepAttnMISL introduces K‐Means clustering for instance‐level features before applying global attention pooling.^[^ ^8^ ^]^ Despite these advances, such methods often fall short of fully leveraging patch‐level features to accurately represent slide‐level features, particularly in capturing the spatial information and interactions between patches within the entire view.

In a broader context, cancer prognosis is not solely determined by histopathology, which often requires the integration of diverse modalities such as clinical information, genomics, and transcriptomics for a comprehensive assessment. Existing research suggests that AI‐based multimodal fusion models can effectively capture multifaceted tumor information, leading to more accurate predictions of patient prognosis.^[^ ^9^, ^10^ ^]^ However, one major obstacle faced when integrating and fusing multi‐modal data is the distinct feature spaces of different modalities which hinder the discovery of cross‐modal correspondence. More importantly, in the real‐world, patients often lack the appropriate genetic sequencing information. Despite the impressive performance of multimodal fusion models, they may not have practical clinical applications due to the absence of complete molecular data in real‐world patient scenarios.

In this study, we developed a bridged multimodal fusion model (Brim) to integrate the WSIs features and genomic molecular features for predicting the prognostic outcomes of pan‐cancer patients. A Transformer‐based MIL method was used to learn the spatial distribution and interactions between patches, incorporating more comprehensive tumor microenvironment information into the model. Meanwhile, a bridge network was designed to learn the associations between paired WSIs and genomic molecular features, especially for predicting missing molecular information using only WSIs. We also performed model interpretation analyses to identify the critical image and molecular features that related to cancer prognosis in pan‐cancers. Furthermore, a validation analysis was performed with an in‐house Colorectal Cancer (CRC) cohort with multimodal datasets from the ICGC‐ARGO project, which provided additional support for the results. Our results demonstrated that the Brim model not only integrated genomic molecular features and WSIs for prognosis prediction but also enabled a WSIs‐only prognosis prediction, which allows a more precise low‐financial burden prognosis prediction in clinical utilization and enables individualized clinical decision‐making.

## 2 Results

### 2.1 A Bridged Multimodal Fusion Model for Prognostic Prediction

**Figure 1:**
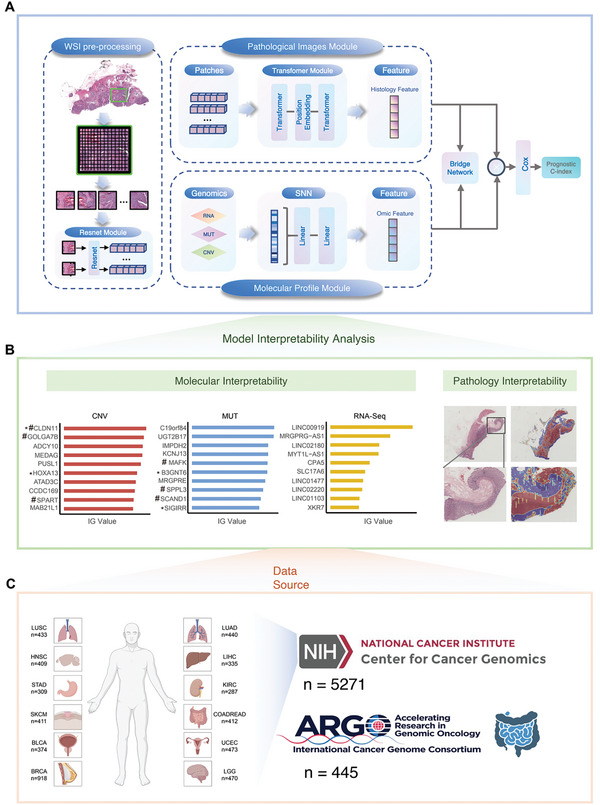
Study Design. A) Overview of the Brim framework. B) Model Interpretability and Visualization. C) Study Cohorts.

To develop clinically applicable multimodal fusion biomarkers, we propose a bridged multimodal fusion model. This model predicts and interprets cancer survival risk by integrating whole slide images (WSIs) with multi‐omics features, including mutation status, copy number variation, and RNA‐seq gene expression. Additionally, it reduces clinical application costs by generating pseudo‐omics embeddings. **Figure** **1**A shows the architecture of Brim. The Brim model consists of four major components: WSI pre‐processing, pathological image profile module, molecular profile module, and bridge network. We first segmented all WSIs using default parameters through the public CLAM repository to extract all image patches, after which we acquired a 1024‐dimensional feature embedding from each image patch by a pre‐trained ResNet50 model.^[^ ^11^ ^]^ To comprehensively learn the spatial heterogeneity of the tumor microenvironment in pathological images, we applied a transformer‐based multiple‐instance learning method^[^ ^12^ ^]^ for integrating the patch‐ and spatial‐level information of WSIs. For the high‐dimensional molecular data, we learned omics features by Self‐Normalizing Networks (SNN) to resist overfitting.^[^ ^13^ ^]^ Additionally, we proposed a bidirectional bridged network that links histology and omics features^[^ ^14^ ^]^ to guarantee the appropriate alignment of feature spaces across different modalities by mapping the paired features into a closely related semantic space. Finally, the fused embeddings were used to predict the survival prognosis of pan‐cancer patients. We also applied the self‐attention visualization and Integrated Gradient (IG) attribution analyses to uncover both WSIs and genomic molecular features that are mostly related to cancer prognosis (Figure 1B). The normalized self‐attention scores of the WSI patches to the predicted risk scores were used to highlight the important image regions that related to patient prognosis. Besides, the calculated absolute integrated gradient scores of all genomic molecular features were used to identify the critical features in prognosis prediction of pan‐cancer patients.^[^ ^15^ ^]^

For model training and performance evaluation, we collected H&E diagnostic WSIs, genomic molecular sequencing data, and clinical information from the TCGA database,^[^ ^16^ ^]^ comprising 5271 pan‐cancer patients across 12 cancer types. Additionally, we also involved an in‐house cohort of colorectal cancer patients named COCC (Clinical Genomic Study of Colorectal Cancer in China) from the ICGC‐ARGO project (https://www.icgcargo.org/), encompassing 445 colorectal cancer patients with complete WSI, molecular sequencing data, and 5‐year clinical follow‐up information (Figure 1C). For patients with multiple WSIs, one WSI was randomly selected as input. All genomic and transcriptomic data were filtered to remove missing features. To ensure a fair comparison between Brim and other models, we utilized five‐fold cross‐validation and identical hyperparameters to train and assess the models' performance. In each training iteration, patient cases from each cohort were randomly divided into non‐overlapping training sets (80%) and validation sets (20%).

### 2.2 Evaluation of Model Performance in Multimodal Data

We initially assessed the performance of Brim in predicting cancer patient prognosis using a five‐fold cross‐validation on the TCGA dataset, utilizing multi‐modal data as input. We compared the performance advantage of the multimodal model with three models trained solely on single‐modal data. Among them, the Transformer‐based Correlated Multiple Instance Learning (TransMIL) model^[^ ^12^ ^]^ and the Attention‐based Deep Multiple Instance Learning (AMIL) model^[^ ^7^ ^]^ were trained exclusively using WSIs, while the Self‐Normalizing Network (SNN) model^[^ ^13^ ^]^ was trained solely based on genomic molecular data. To gauge the prognostic performance of each model, we employed the average concordance index (C‐index) from cross‐validation to measure the accuracy of each model.

Compared to all single‐modal models, Brim demonstrated the highest overall average C‐index across all 12 cancer types at 0.682. In contrast, the average C‐index of TransMIL, AMIL, and SNN was 0.621, 0.620, and 0.650, respectively. Although TransMIL achieved a slightly higher C‐index than Brim in LUSC (TransMIL C‐index 0.596, Brim C‐index 0.595), Brim exhibited the highest C‐index performance in all other cancers. Specifically, Brim achieved a C‐index of 0.677 (95% confidence interval (CI) 0.604 to 0.749) in BRCA and 0.720 (95% CI 0.681 to 0.759) in COADREAD, which established an average improvement of ≈10% compared to the other unimodal models. Additionally, Brim achieved the highest C‐index of 0.838 (95% CI 0.819 to 0.858) in TCGA‐LGG, an improvement of ≈17% compared to TransMIL (**Table** **1**).

**Table 1:** C‐index Model Performances on Survival Prediction across 12 Cancer Types.

TCGA‐BLCA	TCGA‐BRCA	TCGA‐COADREAD	TCGA‐HNSC	TCGA‐KIRC	TCGA‐LGG	TCGA‐LIHC	TCGA‐LUAD	TCGA‐LUSC	TCGA‐SKCM	TCGA‐STAD	TCGA‐UCEC	Overall
SNN												
0.653 (0.597‐0.709)	0.613 (0.553‐0.673)	0.623 (0.586‐0.660)	0.619 (0.575‐0.663)	0.684 (0.611‐0.757)	0.812 (0.777‐0.847)	0.651 (0.612‐0.690)	0.635 (0.601‐0.670)	0.569 (0.529‐0.610)	0.647 (0.615‐0.678)	0.608 (0.573‐0.643)	0.689 (0.633‐0.745)	0.650
AMIL												
0.583 0.542‐0.623)	0.617 (0.577‐0.657)	0.615 (0.555‐0.675)	0.576 (0.551‐0.602)	0.634 (0.592‐0.675)	0.705 (0.623‐0.787)	0.687 (0.645‐0.728)	0.602 (0.564‐0.639)	0.569 (0.516‐0.622)	0.607 (0.550‐0.663)	0.599 (0.538‐0.660)	0.650 (0.582‐0.718)	0.620
TransMIL												
0.624 (0.595‐0.652)	0.616 (0.570‐0.662)	0.652 (0.625‐0.680)	0.592 (0.568‐0.615)	0.639 (0.596‐0.683)	0.669 (0.620‐0.718)	0.640 (0.594‐0.685)	0.606 (0.565‐0.646)	0.596 (0.569‐0.622)	0.580 (0.538‐0.623)	0.591 (0.553‐0.628)	0.643 (0.590‐0.695)	0.621
MMF												
0.653 (0.605‐0.701)	0.651 (0.607‐0.695)	0.709 (0.654‐0.763)	0.623 (0.609‐0.638)	0.675 (0.611‐0.757)	0.837 (0.822‐0.852)	0.682 (0.634‐0.730)	0.643 (0.610‐0.677)	0.591 (0.539‐0.643)	0.641 (0.615‐0.667)	0.619 (0.573‐0.666)	0.714 (0.685‐0.742)	0.670
Brim												
0.666 (0.620‐0.712)	0.677 (0.604‐0.749)	0.720 (0.681‐0.759)	0.633 (0.607‐0.659)	0.692 (0.612‐0.773)	0.838 (0.819‐0.858)	0.690 (0.635‐0.745)	0.647 (0.605‐0.689)	0.595 (0.545‐0.645)	0.660 (0.632‐0.659)	0.640 (0.624‐0.658)	0.723 0.706‐0.739)	0.682

**Figure 2:**
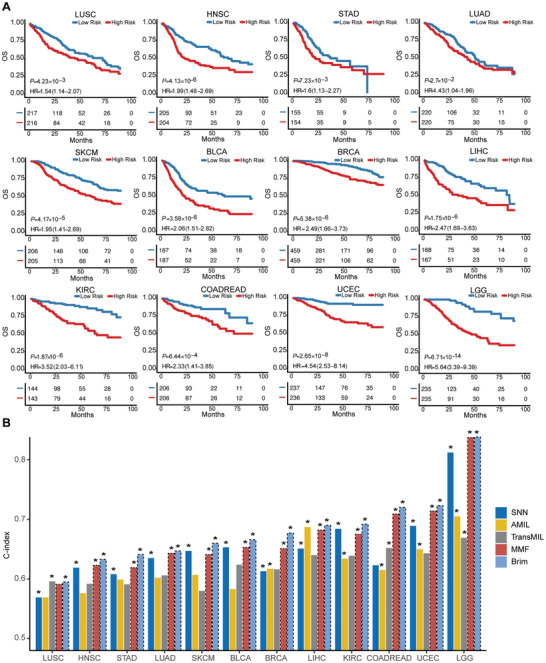
Performance comparison of Brim and baseline methods in prognosis prediction. A) Kaplan–Meier analysis of patients stratified into low‐ and high‐risk groups based on the median predicted risk scores of the Brim model across all 12 cancer types, statistical significances were assessed using the log‐rank test (*p* < 0.05). B) Comparisons of the C‐indexes of SNN, AMIL, TransMIL, MMF, and Brim models in each cancer type, the bar plots represent the mean values of five‐fold cross‐validation, the black dashed bars represent multimodal fusion models and the rest represent unimodal models.^*^ Represents significance of Kaplan–Meier analysis.

We further employed Kaplan–Meier survival curves to visually represent the stratification quality of models’ predictions for high‐risk and low‐risk patients. Log‐rank tests were conducted to examine the statistical differences between the high and low‐risk groups. Overall, Brim achieved statistically significant risk stratification across all 12 cancer types, demonstrating its high reliability in prognosis prediction (**Figure** **2**). In contrast, AMIL could significantly stratify high‐ and low‐risk patients in only 6 out of the 12 cancers, while TransMIL could do so in only three cancers (Figures S1 and S2, Supporting Information). Compared to SNN models relying exclusively on genomic molecular data, Brim demonstrated statistically significant improvements in the majority of cancers (10 out of 12), with slightly lower significance observed in LUSC and LUAD cancers (Figure S3, Supporting Information). These findings suggest that Brim's integration of multiple modalities offers a promising approach to enhancing the accuracy of cancer prognosis predictions over single‐modal models.

### 2.3 Evaluation of the Model Framework in Cancer Representation Learning

After demonstrating the superior performance of Brim compared to state‐of‐the‐art single‐modal models, we conducted a comparative analysis with an established multimodal fusion model (MMF).^[^ ^17^ ^]^ The overall average C‐index of MMF across the 12 cancers was 0.670, lower than Brim's 0.682 (Table 1). Moreover, the performance of Brim surpassed that of MMF in all individual cancers (Figure 2). Additionally, Brim successfully stratified high‐risk and low‐risk patients in LUSC, a task that MMF could not achieve (hazard ratio (HR) 1.27, 95% CI 0.94‐1.71, *p* > 0.05) (Figure S4, Supporting Information). These results further affirm that Brim can better integrating information from different modalities.

To gain a deeper understanding of the bridge module's contribution to semantic learning in multi‐omics data for the prognosis task, we conducted additional experiments that evaluated the Brim model against both a later concatenated model and a single‐directional model, which inferred pathology‐to‐molecular embeddings Our results demonstrated that the Brim model achieved a higher C‐index across all cancer subtypes compared to the later concatenated model (non‐Bridge), and outperformed the single‐directional model in 11 out of 12 cancer subtypes, except for TCGA‐LUSC (Table S1, Supporting Information). In addition, to leverage recent advancements in large visual models (LVMs) for pathology analysis, we incorporated whole‐slide image (WSI) embeddings learned from foundational models such as UNI^[^ ^18^ ^]^ as pathology encoders. We compared the Brim model with two slide‐level encoders based on UNI, one utilizing average pooling and the other employing Attention‐based Deep Multiple Instance Learning (ABMIL) for information fusion. The comparison of C‐index scores across different models revealed that the Brim model outperformed the others in 7 out of 12 cancer subtypes, while the UNI‐ABMIL model led in 2 subtypes, and the UNI‐AVG model led in three subtypes (Table S2, Supporting Information). In conclusion, these findings demonstrate that the bridge framework within Brim effectively aligns multi‐omics data and enhances the representation of learning for cancer prognosis.

### 2.4 Evaluation of Model Performance with Only WSIs

While Brim exhibits excellent performance in predicting the prognosis of pan‐cancer patients using multimodal data, the practical application of using multimodal data as input in a clinical setting is severely hindered due to the challenges of obtaining paired genomic sequencing data from patients in the real‐world. To address that, the Brim model was designed to leverage WSIs to simulate pseudo‐genomic molecular features, enabling high‐dimensional multimodal fusion for prognosis prediction when only WSIs are available (Figure S5, Supporting Information). This approach significantly enhances the model's practical applicability. To assess the semantic alignment of inferred WSI embeddings with their corresponding molecular profiles, we evaluated the mutual information (MI) score and Jaccard index between k‐nearest neighbor (k‐NN) graphs of the inferred WSI embeddings from Brim and TransMIL, and the paired inferred molecular embeddings of Brim. Our results showed that the WSI embeddings inferred by the Brim model achieved higher MI scores and Jaccard indices across cancer subtypes (Figure S6, Supporting Information), highlighting the improved semantic alignment and cross‐modality learning of the Brim model.

Additionally, we compared the performance of Brim and TransMIL in predicting prognosis using only WSIs. Brim achieved the highest overall average C‐index (0.630) across all 12 cancer types, surpassing the average C‐index of TransMIL (0.621). Specifically, except for BLCA and LUSC, Brim outperformed TransMIL in all cancers. Notably, Brim's average C‐index in BRCA and KIRC was 0.631 (95% CI 0.587‐0.676) and 0.654 (95% CI 0.606‐0.703), respectively, surpassing TransMIL by 1.5%. In UCEC, Brim achieved the highest C‐index of 0.683 (95% CI 0.643–0.724) (**Table** **2**). These results confirm the practical value of the Brim model in specific clinical scenarios.

**Table 2:** C‐index Model Performance Comparison between Brim (missing genomic data) and TransMIL.

TCGA‐BLCA	TCGA‐BRCA	TCGA‐COADREAD	TCGA‐HNSC	TCGA‐KIRC	TCGA‐LGG	TCGA‐LIHC	TCGA‐LUAD	TCGA‐LUSC	TCGA‐SKCM	TCGA‐STAD	TCGA‐UCEC	Overall
TransMIL												
0.624 (0.595‐0.652)	0.616 (0.570‐0.662)	0.652 (0.625‐0.680)	0.592 (0.568‐0.615)	0.639 (0.596‐0.683)	0.669 (0.620‐0.718)	0.640 (0.594‐0.685)	0.606 (0.565‐0.646)	0.596 (0.569‐0.622)	0.580 (0.538‐0.623)	0.591 (0.553‐0.628)	0.643 (0.590‐0.695)	0.621
Brim (Missing Genomic data when inferring)												
0.620 (0.590‐0.649)	0.631 (0.587‐0.676)	0.670 (0.593‐0.747)	0.598 (0.550‐0.646)	0.654 (0.606‐0.703)	0.670 (0.593‐0.747)	0.648 (0.617‐0.679)	0.614 (0.591‐0637)	0.576 (0.552‐0.600)	0.591 (0.559‐0.623)	0.605 (0.574‐0.637)	0.683 (0.643‐0.724)	0.630

### 2.5 Model Interpretability Analysis

**Figure 3:**
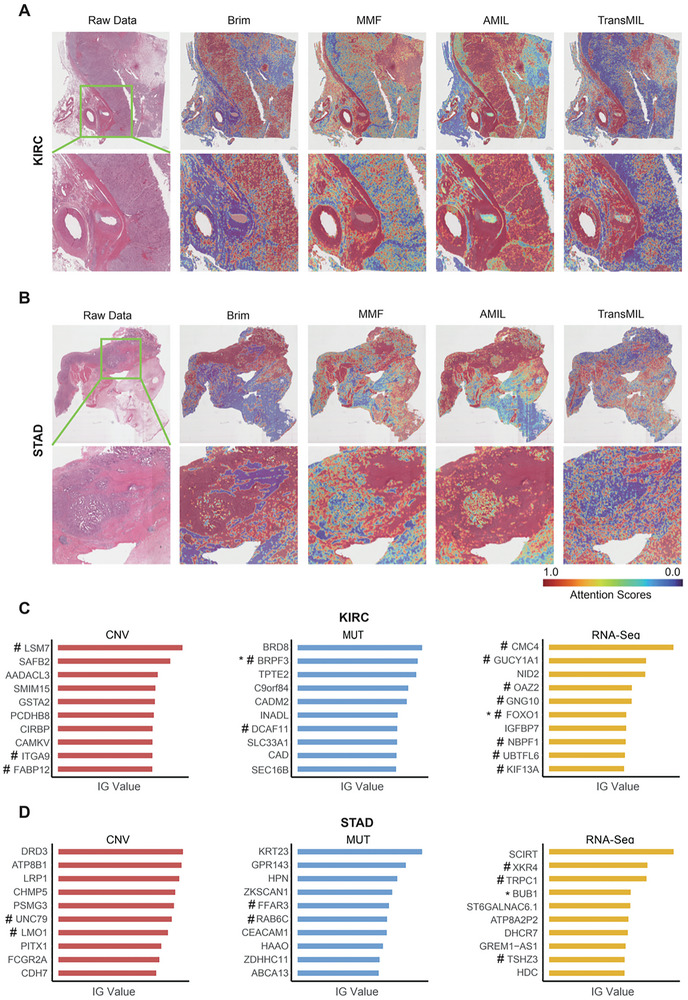
Model interpretability and visualization of prognosis‐related features. A) Represent WSI plots of AMIL, TransMIL, MMF, and Brim models in the KIRC cohort, high‐attention regions in the heatmaps of represent WSI plot correspond to highly contributed morphological features in prognosis prediction. B) Plots as in (A) within the STAD cohort. C) The calculated IG values of individual genomic molecular features based on the trained Brim model within the KIRC cohort, ^*^ represents the identified genomic molecular features with literature validation, and # represents features with statistical significance in univariate Cox regression analysis (*p* < 0.05). D) Plots as in (C) within the STAD cohort.

To enhance the clinical plausibility of our model and ensure a robust biological basis, we employed interpretable approaches such as attention and integrated gradient (IG) attribution analyses to characterize the contributions of WSI patches and genomic molecular features across TransMIL, AMIL, MMF, and Brim models in pan‐cancer prognosis prediction. For WSI plots, we calculated attention scores for each WSI and generated high‐resolution attention heatmaps to highlight the critical regions that mostly contributed to the prognostic prediction. Our analysis revealed that Brim exhibited a greater focus on tumor cell regions in KIRC and STAD but not with the TransMIL, AMIL, and MMF, suggesting Brim can capture more relevant biological information in pathological images (**Figure** **3**A). To investigate the impact of different genomic molecular features in prognostic prediction, we then assessed their contributions to the predicted risk scores by calculating their IG values. Specifically, we selected the top ten genes with the highest IG values in copy number variation (CNV), mutation, and gene expression datasets to uncover the biological relationships in model prognosis prediction (Figure 3B). To further support our conclusions, we conducted a comprehensive search of these genomic molecular features in NCBI and other databases and examined their independent relationship with disease prognosis using univariate Cox regression analysis (Table S3, Supporting Information). In KIRC, the IG analysis has revealed two genes with known roles in patient survival outcomes and thirteen genes with significant prognostic contributions.^[^ ^19^, ^20^ ^]^ Similarly, in STAD, one gene with known relevant roles and seven genes with significant prognostic contributions were identified.^[^ ^21^ ^]^ We then extended these analyses to other cancers and confirmed that Brim can robustly focus more on tumor regions in WSIs and uncovered genomic molecular features that are highly associated with patient prognosis (Figures S7, S8, S9, S10 and S11, Supporting Information).

### 2.6 Validation of Model Performance in COCC

**Figure 4:**
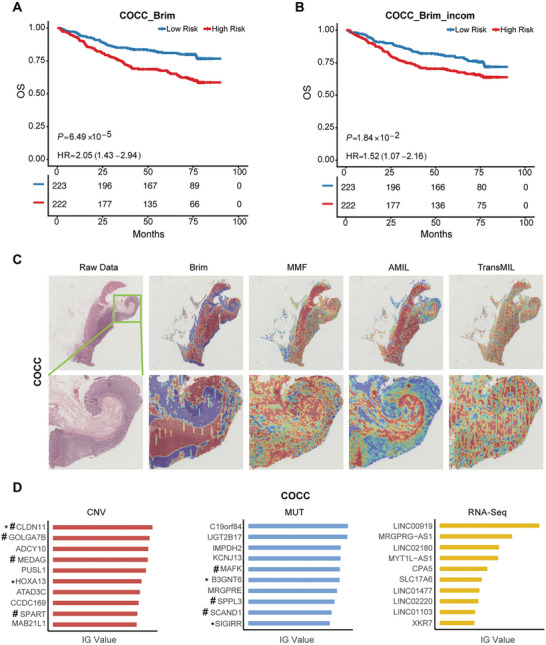
Model validation with the in‐house COCC Cohort. A) Kaplan–Meier analysis of patients stratified into low‐ and high‐risk groups based on the predicted risk scores of the Brim model in the COCC cohort with complete multimodal datasets. B) Kaplan–Meier analysis as in (A) with incomplete multimodal data (missing genomics). C) Represent WSI plots of AMIL, TransMIL, MMF, and Brim models in the COCC cohort, high‐attention regions in the heatmaps of represent WSI plot correspond to high contributed morphological features in prognosis prediction. D) The calculated IG values of individual genomic molecular features based on the trained Brim model within the COCC cohort, ^*^ represents the identified genomic molecular features with literature validation, and # represents features with statistical significance in univariate Cox regression analysis (*p* < 0.05).

To further validate the reliability of our model and results, we conducted an independent validation analysis using an in‐house CRC cohort (COCC) (Table S4, Supporting Information). Through this validation cohort, we demonstrated that Brim accurately stratifies high‐risk and low‐risk patients in the COCC cohort when provided with multimodal data (HR 2.05, 95% CI 1.43–2.94, *p* < 0.0001) (**Figure** **4**A), outperforming other models (Figure S12, Supporting Information). Importantly, Brim achieved the same results when only WSIs were available as input (HR 1.52, 95% CI 1.07–2.16, *p* < 0.05) (Figure 4B). To ensure the consistency of our model, we conducted the interpretability analysis in the COCC cohort as described before. The attention heatmap analysis revealed that Brim can exhibit a strong focus on regions of WSI‐containing tumor cells in the COCC cohort, but not with other models (Figure 4C). We also identified the critically prognostic genomic molecular features with our model in the COCC cohort, within the top ten genomic molecular features selected by IG values, we found that the *CLDN11*, *HOXA13*, *B3GNT6*, and *SIGIRR* genes were reported to be associated with the progression of colorectal cancer.^[^ ^22^, ^23^, ^24^, ^25^, ^26^, ^27^, ^28^ ^]^ Additionally, we discovered that *CLDN11, GOLGA7B, MEDAG, SPART, MAFK, SPPL3, and SCAND1* were significantly associated with the prognosis of colorectal cancer by univariate Cox regression analysis (Figure 4D). The validation results in our in‐house cohort were consistent with our previous findings and provided extra evidence to support the capability of Brim to accurately predict patient prognosis using both the complete modalities and WSI‐only inputs. Meanwhile, the ability of Brim to achieve consistent results across different ethnic cohorts highlights its versatility and robustness as a robust clinical decision‐making tool.

## 3 Conclusion

The complexity and heterogeneity of cancers often result in unpredictable clinical outcomes and low treatment response. Accurately prognosis predicting is crucial for making targeted clinical decisions and achieving precise treatment of cancer patients. However, current cancer staging systems, such as TNM staging, are still unable to accurately predict the clinical outcomes of individual patients due to the insufficient account for tumor heterogeneity.^[^ ^29^ ^]^ Therefore, new methods that can accurately predict prognosis by taking into account as much tumor information as possible are urgently needed. The prognostic prediction of cancer has been extensively investigated using data from WSIs, radiomics, and genomics.^[^ ^30^, ^31^, ^32^ ^]^ However, relying solely on a single modality cannot fully characterize the heterogeneity in tumor tissues, leading to some overlooked information that affects patient prognosis prediction. To overcome that, multiple multimodal learning models were developed to integrate multiple datasets of tumor tissues with different scales and achieve higher prognosis prediction performance.^[^ ^17^, ^33^, ^34^ ^]^ However, the limited and infeasible paired multimodal datasets in clinical applications severely hindered the usage of these methods, which requires a more flexible framework to allow for learning cross‐modality relationships and missing modality predicting.

In this study, we developed a bridged multimodal fusion model named Brim. The Brim model comprises a pathological image module, a genomic molecular module, and a bridge network that learns and integrates the associations between WSIs and genomic molecular features to predict survival outcomes in pan‐cancer patients. The pathological image module utilizes a Transformer‐based multi‐instance learning method^[^ ^12^ ^]^ to integrate the plaque and spatial‐level information of WSIs, while the genomic molecular module uses a Self‐Normalizing Network (SNN) ^[^ ^13^ ^]^ for integrating and extracting the CNV, mutation, and gene expression features in patient tumor tissues. The bridge network was constructed by a bi‐directional autoencoder network to learn cross‐modal interactions.^[^ ^14^ ^]^ The Brim model achieves the best performance in multimodal pan‐cancer prognosis prediction with an average C‐index of 0.682, a general improvement of ≈5% compared to the performance of current state‐of‐the‐art unimodal models, and outperforms the performance of the established multimodal fusion model (MMF).^[^ ^17^ ^]^ Furthermore, with the bi‐directional bridged network, the Brim model enables prognosis prediction with only WSIs, achieved an average C‐index of 0.63 across 12 cancers, and outperformed the WSIs‐only model.

The Brim model offers an optimized WSI feature extraction method that fully considers the spatial location and environment of the pathological tissue to capture higher‐level structural information (e.g., tumor shape or extent) and characterize the tumor heterogeneity. Additionally, it offers a flexible framework for integrating other WSI embedding methods,^[^ ^18^ ^]^ enabling advanced pathology learning. We also interpreted the critical genomic molecular features in the prognosis prediction of all cancer types. With the independent validation CRC cohort (COCC), we demonstrated that the Brim model can robustly predict the prognosis of pan‐cancer patients with different clinical subgroups and ethnic cohorts. Besides, we proved that the missing modality prediction based on learned associations across modalities can extract more information with only WSIs and help prognosis prediction with limited clinical resources.

Although our approach has shown promising results, there are still several limitations. The black‐box nature^[^ ^35^ ^]^ of the deep learning methods makes it challenging to provide a precise biological rationale for the decision‐making process of the Brim model. Moreover, although we demonstrated the improved Brim's predictive accuracy by generating pseudo‐genomic features from WSIs, the underlying biology mechanism represented by these features remains unclear. Further exploration methods to characterize the regulatory associations between WSIs and genomic molecular features are required.

In conclusion, we have developed a bridged multimodal fusion model for prognosis prediction of pan‐cancer patients. The Brim model surpasses existing state‐of‐the‐art methods when complete multimodal modality datasets are available and outperforms existing unimodal models for pathology analysis with only WSIs. This model not only enables clinicians to make more informed decisions but also significantly reduces the cost of clinical prognosis prediction for cancer patients, which is a more practical and effective tool in clinical diagnostic decision‐making

## 4 Experimental Section

### Study Participants

In this study, the H&E diagnostic WSIs, genomic molecular features, and clinical information of pan‐cancer patients were collected from the TCGA database. The inclusion criteria involve: 1) patients with complete 5‐year survival follow‐up information, 2) patients possessing complete corresponding gene expression, copy number variation (CNV), and mutation data, and 3) the selected cancer type should have at least 250 uncensored survival data cases. In total, 12 cancer types and 5271 patient samples were involved for pan‐cancer prognosis model training and testing, which range from 287–918 patients in individual cancers. The TCGA projects COAD and READ were combined into a single cohort, TCGA‐COADREAD. All preprocessed data were acquired via the UCSC Xena platform (https://xena.ucsc.edu/) at the University of California, Santa Cruz.

An in‐house cohort of colorectal cancer patients named COCC (Clinical Genomic Study of Colorectal Cancer in China) from the ICGC‐ARGO project (https://www.icgcargo.org/) was also included. All patient samples were obtained from the Sixth Affiliated Hospital of Sun Yat‐sen University, and 445 patient samples with the same inclusion criteria of the TCGA pan‐cancer cohort were selected. Written informed consent was obtained from all anticipated patients and approved by the ethics committees of the Sixth Affiliated Hospital of Sun Yat‐sen University. A detailed summary of the clinical and demographic characteristics of patients in each cohort is presented in **Table** **3**.

**Table 3:** Characteristics of patients in all cohorts.

	TCGA‐BLCA (n = 374)	TCGA‐BRCA (n = 918)	TCGA‐COADREAD (n = 412)	TCGA‐HNSC (n = 409)	TCGA‐KIRC (n = 287)	TCGA‐LGG (n = 470)	TCGA‐LIHC (n = 335)	TCGA‐LUAD (n = 440)	TCGA‐LUSC (n = 433)	TCGA‐SKCM (n = 411)	TCGA‐STAD (n = 309)	TCGA‐UCEC (n = 473)	COCC (n = 445)
	n = 374	n = 918	n = 412	n = 409	n = 287	n = 470	n = 335	n = 440	n = 433	n = 411	n = 309	n = 473	n = 445
Age													
<65years	138 (36.9%)	645 (70.26%)	189 (45.87%)	255 (62.35%)	190 (66.2%)	436 (92.77%)	203 (60.6%)	197 (44.77%)	146 (33.72%)	262 (63.75%)	128 (41.42%)	256 (54.12%)	280 (62.92%)
≥65years	236 (63.1%)	273 (29.74%)	223 (54.13%)	154 (37.65%)	97 (33.8%)	34 (7.23%)	132 (39.4%)	233 (52.95%)	282 (65.13%)	149 (36.25%)	178 (57.61%)	215 (45.45%)	165 (37.08%)
Sex													
Female	96 (25.67%)	907 (98.8%)	200 (48.54%)	109 (26.65%)	99 (34.49%)	208 (44.26%)	111 (33.13%)	234 (53.18%)	105 (24.25%)	152 (36.98%)	106 (34.3%)	473 (100%)	191 (42.92%)
Male	278 (74.33%)	11 (1.2%)	212 (51.46%)	300 (73.35%)	188 (65.51%)	262 (55.74%)	224 (66.87%)	206 (46.82%)	328 (75.75%)	259 (63.02%)	203 (65.7%)	0 (0%)	254 (57.08%)
TNM Stage													
I	2 (0.53%)	157 (17.1%)	76 (18.45%)	20 (4.89%)	159 (55.4%)	0 (0%)	157 (46.87%)	239 (54.32%)	210 (48.5%)	66 (16.06%)	36 (11.65%)	0 (0%)	56 (12.58%)
II	118 (31.55%)	529 (57.63%)	138 (33.5%)	59 (14.43%)	30 (10.45%)	0 (0%)	77 (22.99%)	109 (24.77%)	142 (32.79%)	119 (28.95%)	98 (31.72%)	0 (0%)	183 (41.12%)
III	130 (34.76%)	195 (21.24%)	127 (30.83%)	67 (16.38%)	61 (21.25%)	0 (0%)	77 (22.99%)	62 (14.09%)	72 (16.63%)	157 (38.2%)	134 (43.37%)	0 (0%)	109 (24.49%)
IV	122 (32.62%)	19 (2.07%)	56 (13.59%)	221 (54.03%)	35 (12.2%)	0 (0%)	4 (1.19%)	24 (5.45%)	5 (1.15%)	20 (4.87%)	34 (11%)	0 (0%)	95 (21.35%)

### Procedures

All WSIs obtained from scanning pathological tissue sections were stored in SVS format. For patients with multiple WSIs, one WSI was randomly selected as input. Each WSI was automatically fully segmented using default parameters through the public CLAM^[^ ^11^ ^]^ repository, and all 256 × 256 image patches extracted after segmentation were down‐sampled to 40 × magnification, corresponding to 0.25 um px^−1^. After that, A pre‐trained ResNet50 model was applied to extract a 1024‐dimensional feature embedding from each image patch. For WSI embedding inference using a large foundation model, the patch encoder, and the pre‐trained weights of the UNI model (https://github.com/mahmoodlab/UNI) were employed. The patch embeddings were then inferred following the approach outlined previously.

To retain genomic molecular information as much as possible, only genetic features with missing values were filtered. The filtered genetic datasets were standardized using the sci‐kit‐learn (version 1.3) package from Python (version 3.7.7).

### Evaluation of Model Performance in Prognosis Prediction

The prognostic accuracy of deep learning models in predicting the overall survival status of pan‐cancer patients was evaluated. The Scikit‐learn package (https://scikit‐learn.org) to perform five‐fold cross‐validation evaluations for characterizing the model performance in each cohort and applied C‐index as a metric for accessing the performance of model prediction, as well as the mean and confidence interval of the C‐index in cross‐validation evaluations. During the evaluation iteration of individual cancers, 80% of patient samples were used as the training set, while the remaining 20% were set as the test dataset for model evaluation.

### Evaluation of Semantic Alignment Across Embeddings

The mutual information between pairs of embeddings was computed using the “ee.mi” function from the “npeet” library. This metric quantifies the amount of information one embedding set provides about another. To assess the similarity between embeddings, the Jaccard Index was employed to compare two graphs derived from the embeddings. K‐Nearest Neighbors (KNN) graphs were constructed using the “build_knn_graph” function, with k = 50 and “cosine” distance as the similarity metric. A higher Jaccard Index indicates greater similarity between the graph structures, suggesting that the embeddings capture similar local relationships.

### Statistical Analysis

Bar graphs represent the mean values obtained through five‐fold cross‐validation. For categorical variables, Chi‐squared tests were used to evaluate differences between groups. When the expected frequency was less than five, Fisher's exact test was applied to assess group differences. For continuous variables, Student's *t*‐test was used to analyze differences between two groups with normal distribution. For groups with non‐normal variance distributions, the Mann‐Whitney U test was employed to determine differences. Kaplan–Meier analysis and log‐rank test were used to assess the statistical significance in stratifying patients with different clinical risks, and the median cutoff value of continuous risk scores was selected. Univariate analysis was performed using the Cox proportional hazards regression model. All analyses were considered statistically significant if the two‐sided *P*‐value was less than 0.05. All statistical analyses were performed with R (version 4.1.2).

### Ethics Statement

The research scheme was approved by the Ethics Review Committee of the Sixth Affiliated Hospital, Sun Yat‐sen University (2023ZSLYEC‐299). Written informed consent was obtained from all participants.

## Conflict of Interest

The authors declare no conflict of interest.

## Supporting information



Supporting Information

## Data Availability

The public data of this study is available through the UCSC Xena platform (https://xena.ucsc.edu/) at the University of California, Santa Cruz. The in‐house data of this study is available on request to the corresponding author with a signed data access agreement. The de‐identified patient‐level clinical data will be provided upon reasonable request. Sharing of genomics data requires approval for data transfer from the institutional and governmental legal departments of both parties, as well as ethics clearance from the ethics committees of both parties. The source code for the deep learning model is available online (https://github.com/dingjunxiang/Brim)
