# Supplementary material for: Interpretable Multimodal Fusion Model for Bridged Histology and Genomics Survival Prediction in Pan‐Cancer
Source: Adv Sci (Weinh). 2025 Mar 7;12(17):2407060. doi: 10.1002/advs.202407060 (PMC12061278; doi:10.1002/advs.202407060)
Supplement: Supplementary file 1 — Supporting Information [file ADVS-12-2407060-s001.docx]

Supporting Information

**Framework of the Brim Model**

**Transformer-based multiple-instance learning** In this paper, we established a prognostic deep learning model that was adapted from a transformer-based multiple-instance learning method^[1]^ that was initially developed for WSI. Consistent with the vision transformer (ViT) framework, we treated a single WSI as the "sentence" of image patches. Accordingly, we utilized transformer layers to reveal the associations between image patches and take a particular token <cls> as WSI representation for downstream survival prediction. Due to the O(n²) memory and time complexity of the standard self-attention mechanism, which makes it challenging to use for feature extraction in WSI, we employed the Nystrom-attention algorithm^[2]^ to compute the approximate self-attention, as shown in the equation below:


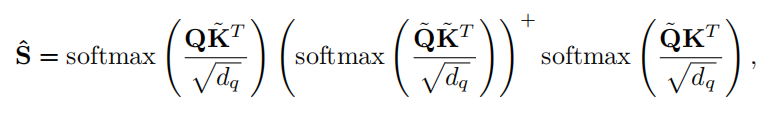
 (1)

Additionally, to learn the spatial location information of image patches, we added a position encoding module to transformer layers, and the resulting WSI features were represented as *Z_path_*.

**Learning genomic feature representations using Self-Normalizing Networks (SNN)** For learning scenarios such as genomics with high-dimensional features and relatively few training samples, the traditional standard feed-forward neural network is easy to overfit and difficult to mine deep abstract representations. We then utilized the self-normalizing neural network (SNN) with scaled exponential linear units (SELU) to prevent the gradient from exploding and the gradient vanishing. The genomic feature representations extracted from the SNN module were then used for survival outcome prediction, represented as *Z_genomic_*.

**Bidirectional bridged network** We proposed a bidirectional bridged network that links pathological and genomic features and maps the features into a closely related semantic space before conducting multimodal fusion for survival analysis. A bidirectional bridged network comprises two fully connected networks, namely the inference network FC_path->genomic_ and the inference network FC_genomic->path_. During model training, the bidirectional bridged network learns a mapping between pathological and genomic features, and the learned associations enable prognosis prediction with missing genomic molecular features.

**Multimodal feature representation fusion for survival prediction** To explicitly explore the interactions between pathological images and genomics, we utilized the Kronecker Product to learn a multimodal representation computed by the vector product of the feature vectors of pathological images and genomics (Z_wsi_ and Z_genomic_), which is shown in the equation below:


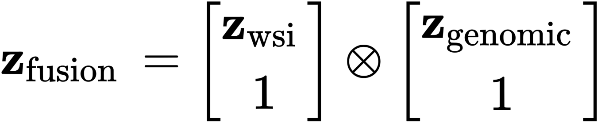
 (2)

Where ⊗ is the vector product, *Z_fusion_* is a special tensor that captures all unimodal as well as multimodal interactions in an element-wise product manner. Later, with the fused representation of multi-modal features as input and the defined Cox object (survival time and survival status)^[3]^ as supervision, Brim learns a fully connected network for survival outcome prediction.

**Brim interpretability and visualization**

Brim interpretability and visualization consist of two components: 1) Self-attention visualization for WSI morphological feature interpretation, and 2) Integrated Gradient attribution for genomic molecular feature interpretation.

**Self-attention visualization for WSI interpretation** To interpret the relative importance of diverse regions of each WSI to patient-level survival outcome prediction of Brim, we computed the self-attention scores of the first transformer encoder and saved the attention scores between <cls> token and all image patches. After that, we applied the SoftMax function to convert the attention scores to a probability distribution. The normalized scores are scaled between zero and one (zero denotes least relative to survival outcome prediction, and vice versa) and transformed into RGB colors. For each WSI attention heatmap, red regions denote high-attention image patches with high prognostic relevance in prognosis prediction, whereas blue regions denote low-attention image patches with low relevance in prognosis prediction.

**Integrated Gradient attribution analysis for genomic interpretation** To explain the importance of molecular features in predicting survival outcomes, we utilize the Integrated Gradient (IG) method^[4]^, which is a gradient-based attribution analysis technique. IG is used to compute the contribution of the input genomic features. IG satisfies two fundamental axioms for attribution analysis: 1) Sensitivity, which states that when some part of the difference between the input and baseline changes, the prediction result of the model also changes, and the attribution map should also be able to express this change. 2) Implementation Invariance, which states that for two networks with the same function, although the structure may not be the same, the attribution map should be the same. In our study, we calculated the IG value using the “Captum” package, with zeros as the baseline feature value. After calculation, we normalized all IG values using the Frobenius norm. Genomic molecular features with high absolute IG values indicate high contributions in the prediction of survival outcomes. For each cohort of different cancer types, we visualized the top 10 highest RNA-Seq abundance features, copy number variation features, and mutation status features, respectively.

**Benchmarking Methods**

Firstly, we evaluated the performance of the Brim model against multiple established deep-learning-based prognostic models^[1, 5-7]^ in 12 cancer types using 5-fold cross-validation with paired WSI-genomic molecular datasets. To assess the predictive ability of the prognostic models, we used the mean cross-validated C-index to compare the agreement of the relative ordering of survival time pairs predicted by the models with actual observations. Additionally, we employed Kaplan-Meier curves to visualize the capability of different models in stratifying low-risk and high-risk patients and used the log-rank test to evaluate the statistical significance of risk patient stratification. Furthermore, we compared the performance of Brim, specifically when only pathological image data is used, with TransMIL, which is a subnetwork of Brim.

**Training details**

In order to fairly compare the performance of the models (Brim, MMF, SNN, TransMIL, and AMIL), we trained and evaluated the performance of models using 5-fold cross-validation and the same hyperparameters. We repeat five times to randomly partition the patient cases of each cohort into non-overlapping training (80%) and validation (20%) sets. The hyperparameters are set as follows:

learning rate: 2 × 10^-3^; optimization: Adam; epoch: 20; batch size: 1.

**Hardware and Software**

The training and inference of all models are accomplished on our computing cluster equipped with eight Nvidia A100 graphics-processing units (GPUs), each possessing 40GB of memory. We acknowledge technical and computing resources support from the Bio-medical big data Operating System (Bio-OS).


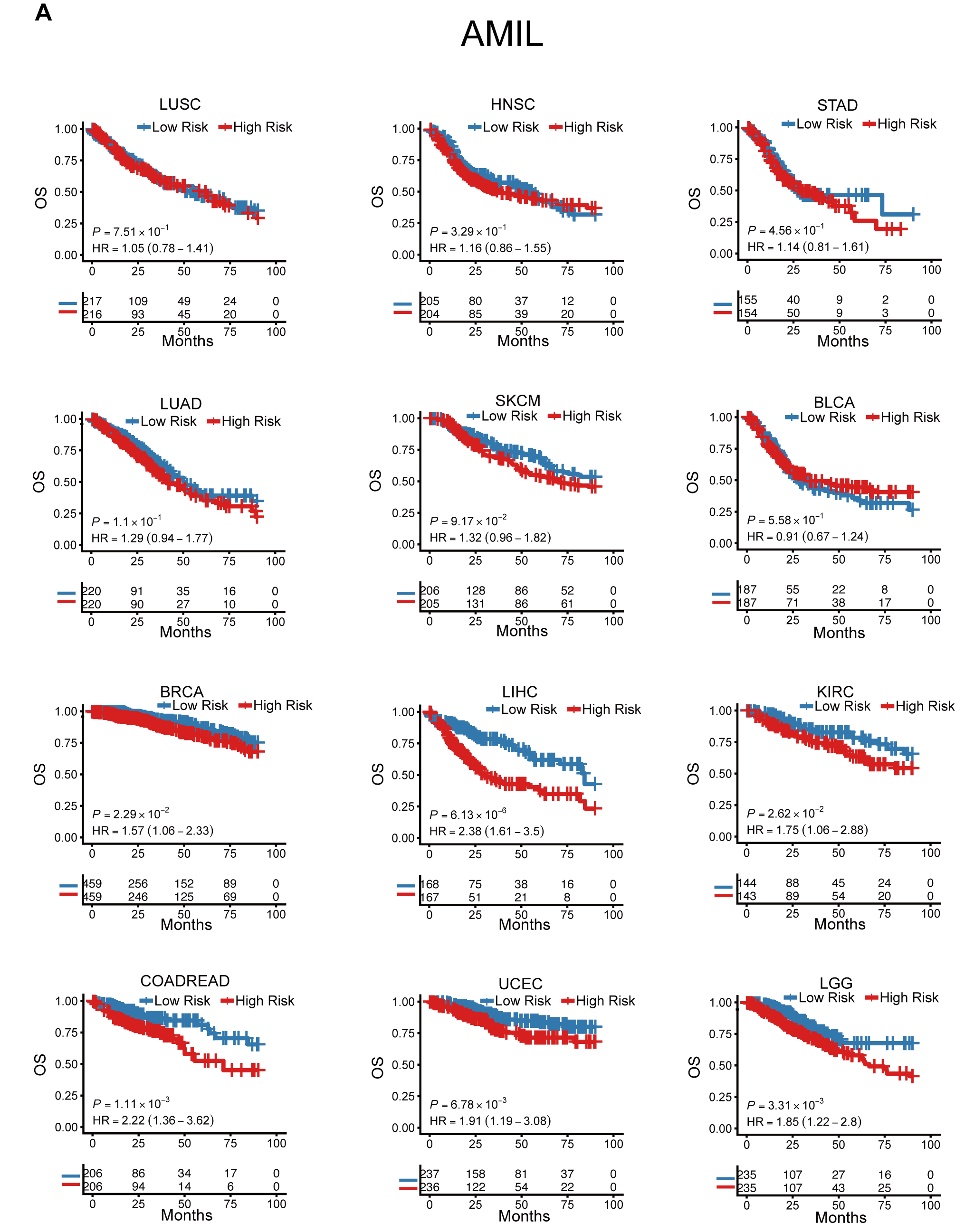


**Figure S1.** Kaplan-Meier Survival Analysis of AMIL

(A) Kaplan-Meier analysis of patients stratified into low and high-risk groups based on the median predicted risks with the AMIL model across all 12 cancer types. The statistical significance of survival distributions between low and high-risk patients was assessed using the log-rank test (*P*<0.05).


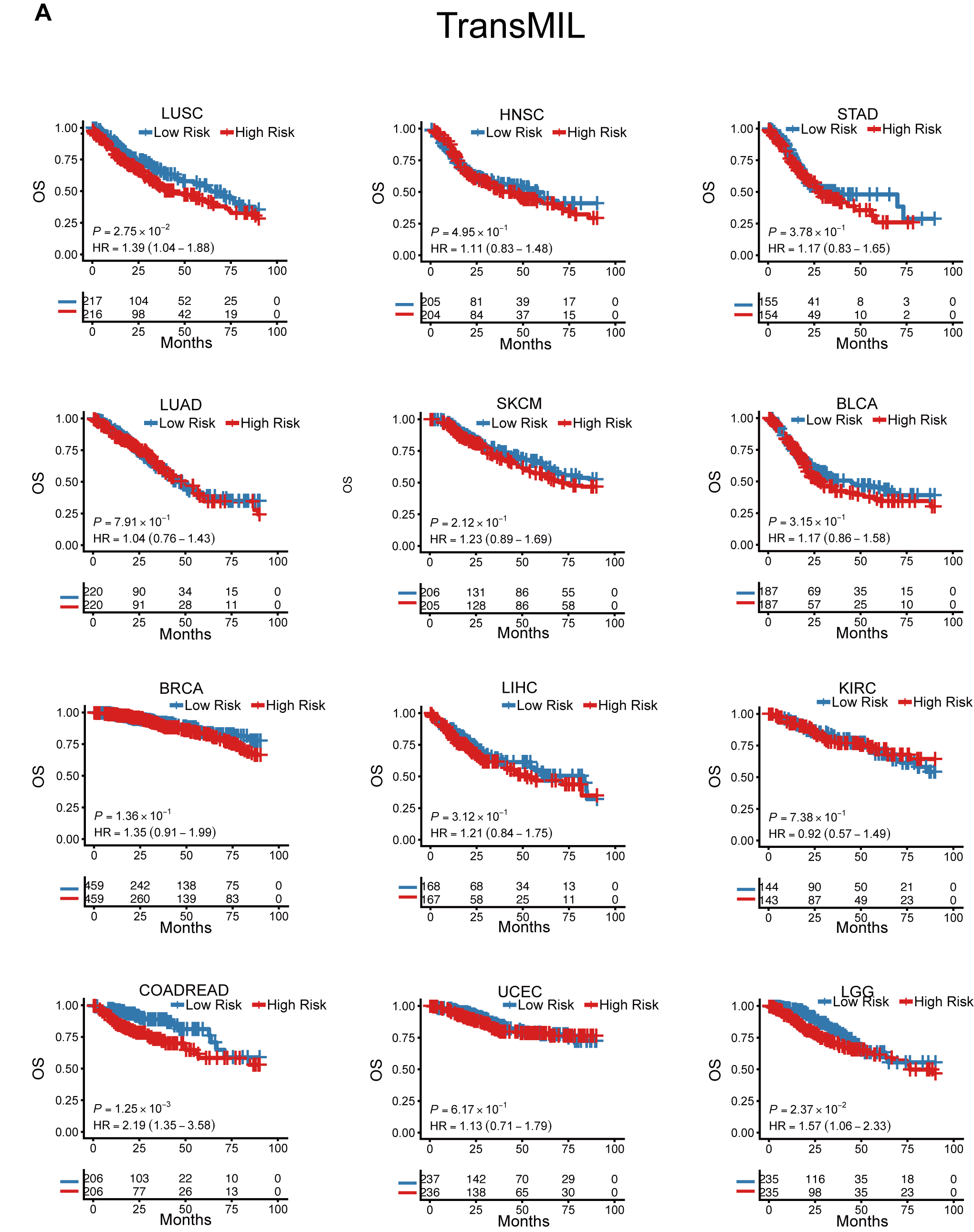


**Figure S2.** Kaplan-Meier Survival Analysis of TransMIL

(A) Kaplan-Meier analysis of patients stratified into low and high-risk groups based on the median predicted risks with the TransMIL model across all 12 cancer types. The statistical significance of survival distributions between low and high-risk patients was assessed using the log-rank test (*P*< 0.05).


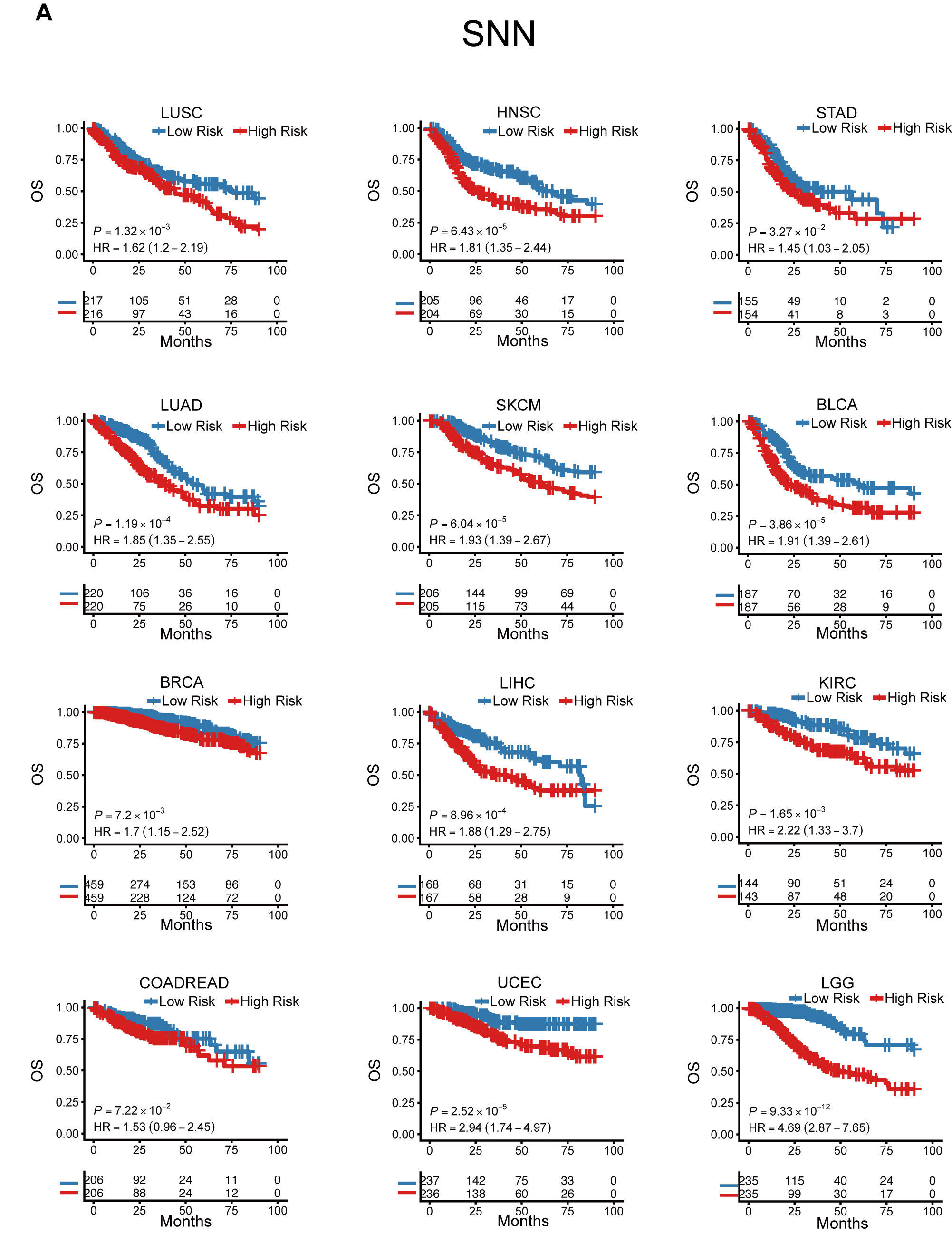


**Figure S3.** Kaplan-Meier Survival Analysis of SNN

1. Kaplan-Meier analysis of patients stratified into low and high-risk groups based on the median predicted risks with the SNN model across all 12 cancer types. The statistical significance of survival distributions between low and high-risk patients was assessed using the log-rank test (*P*<0.05).


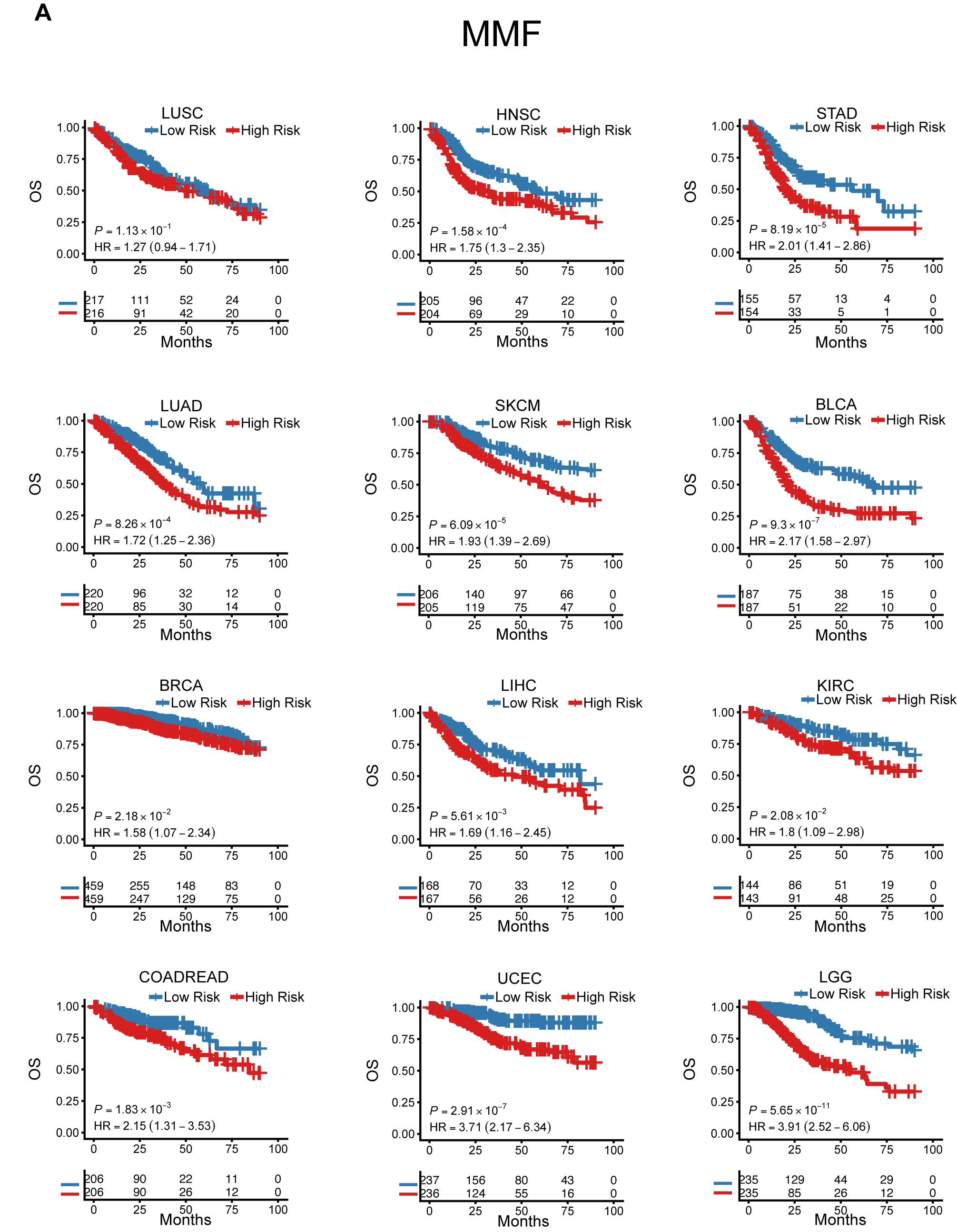


**Figure S4.** Kaplan-Meier Survival Analysis of MMF

(A) Kaplan-Meier analysis of patients stratified into low and high-risk groups based on the median predicted risks with the MMF model across all 12 cancer types. The statistical significance of survival distributions between low and high-risk patients was assessed using the log-rank test (*P*<0.05).


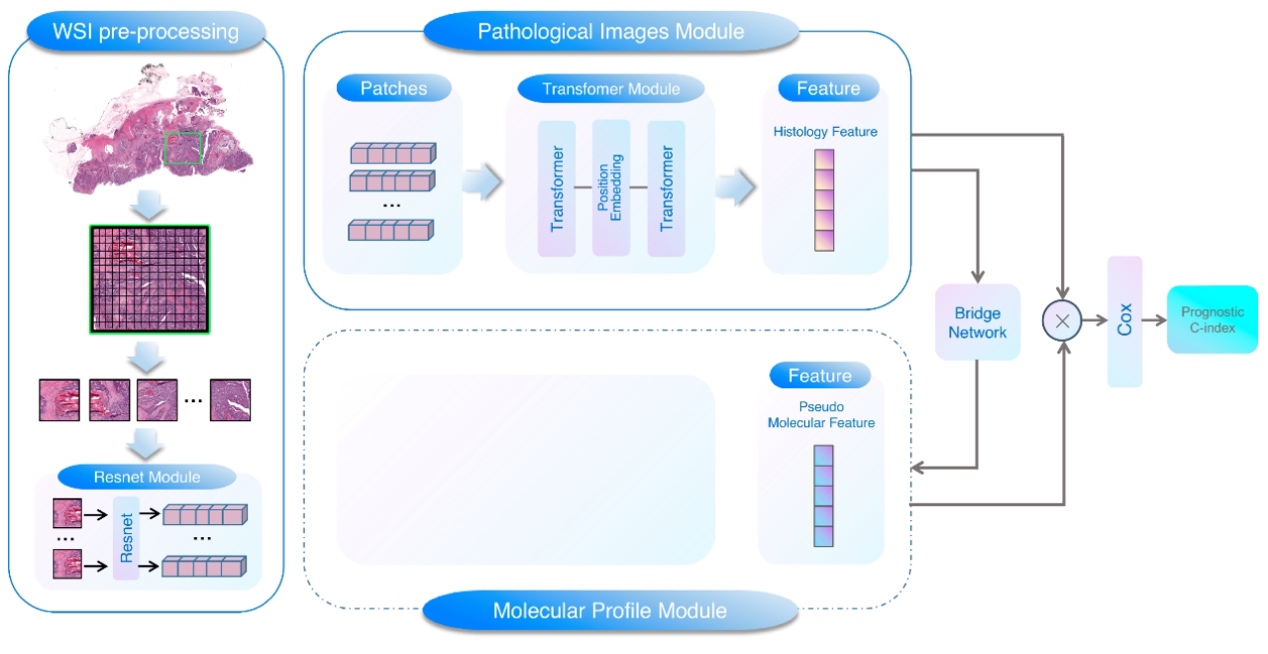


**Figure S5.** Framework of Brim model in the case of missing genomic data prognosis prediction


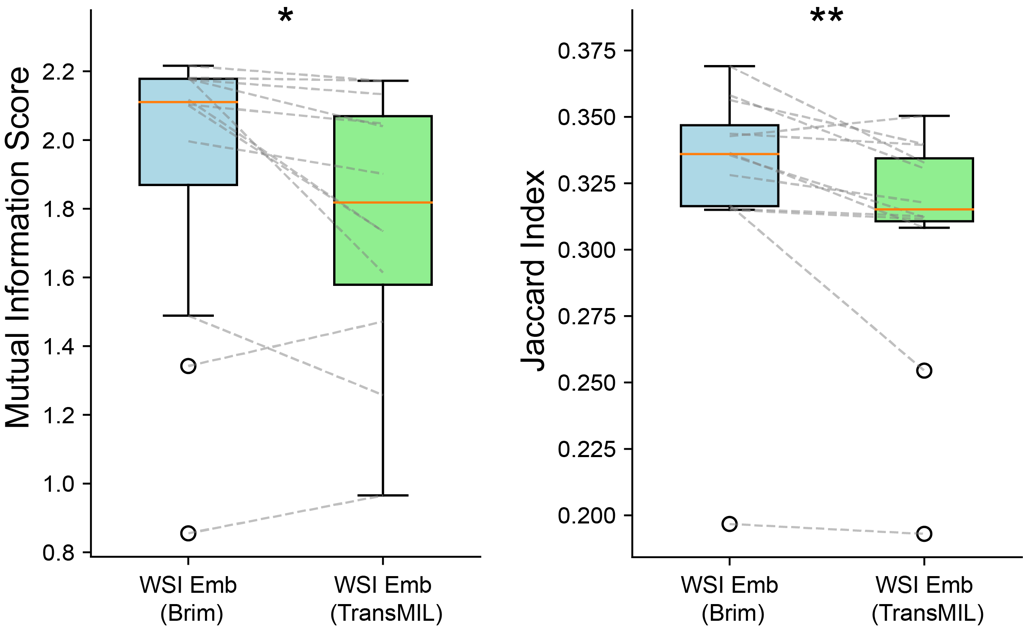


**Figure S6.** Evaluation of semantic alignment across different WSI embeddings with paired molecular profiles. Comparison of the inferred Mutual Information Score (left) and Jaccard Index for WSI embeddings generated by the Brim and TransMIL models, compared to the paired molecular embeddings from the Brim model. Statistical significance was assessed using the paired Mann-Whitney U test, with * indicating p-value < 0.05 and ** indicating p-value < 0.01.


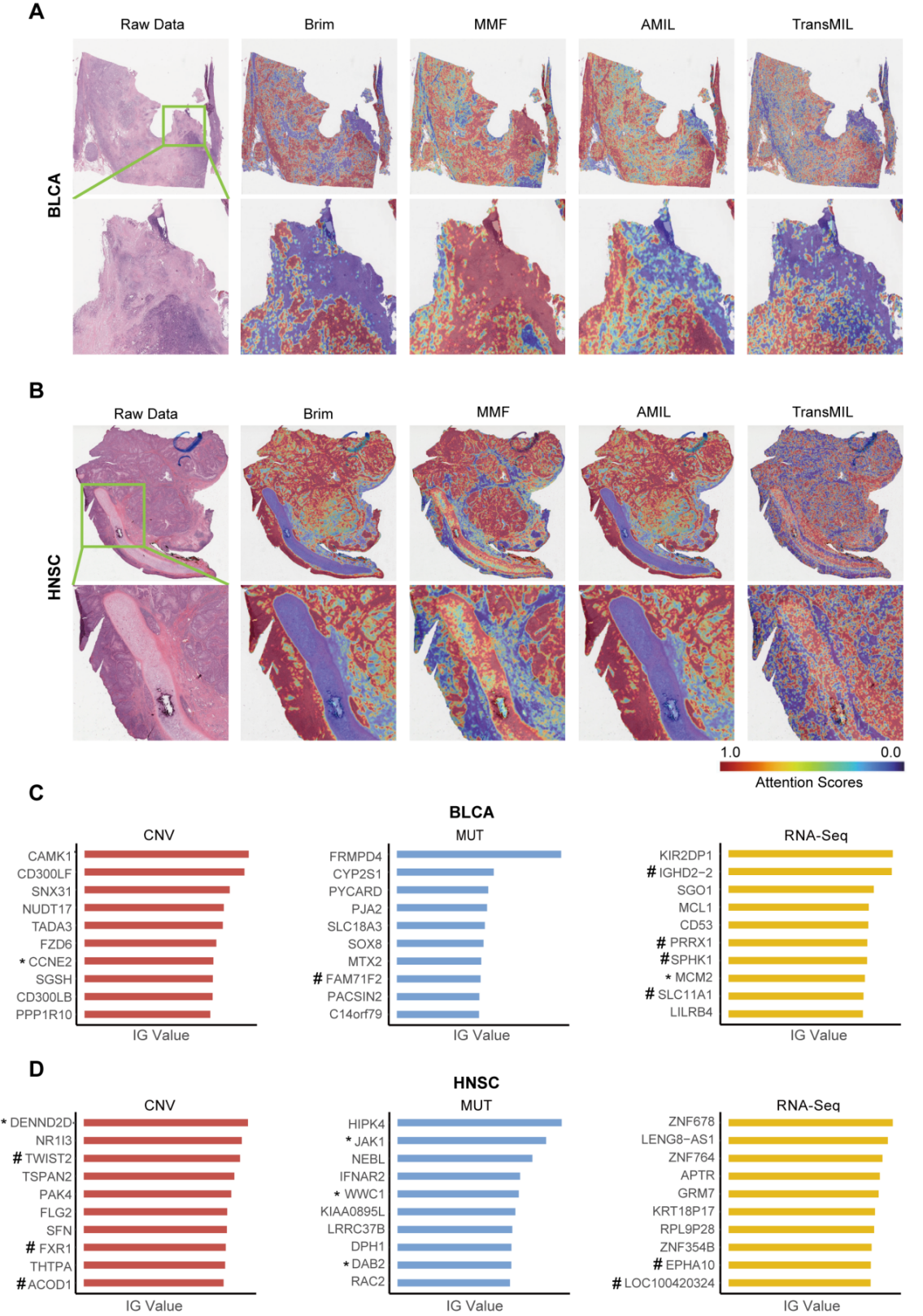


**Figure S7.** Model Interpretability and visualization in BLCA and HNSC

(A) Represent WSI plots of AMIL, TransMIL, MMF and Brim models in BLCA cohort, high-attention regions in the heatmaps of represent WSI plot correspond to high contributed morphological features in prognosis prediction. (B) Plots as in (A) within the HNSC cohort. (C) The calculated IG values of individual genomic molecular features based on the trained Brim model within the BLCA cohort, * represents the identified genomic molecular features with literature validation, and # represents features with statistical significance in univariate Cox regression analysis (*P*<0.05). (D) Plots as in (C) within the HNSC cohort.


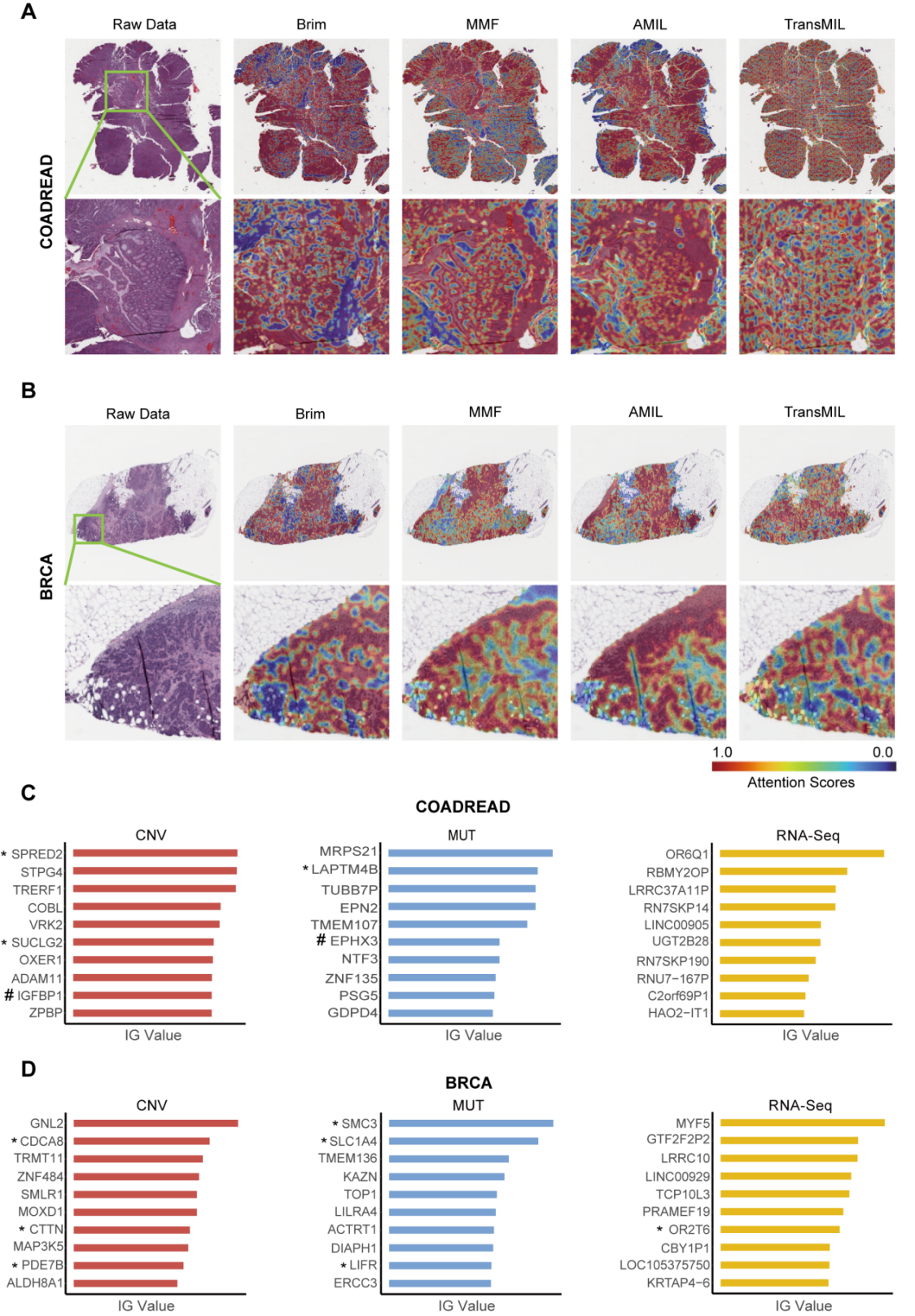


**Figure S8.** Model Interpretability and visualization in COADREAD and BRCA

(A) Represent WSI plots of AMIL, TransMIL, MMF and Brim models in COADREAD cohort, high-attention regions in the heatmaps of represent WSI plot correspond to high contributed morphological features in prognosis prediction. (B) Plots as in (A) within the BRCA cohort. (C) The calculated IG values of individual genomic molecular features based on the trained Brim model within the COADREAD cohort, * represents the identified genomic molecular features with literature validation, and # represents features with statistical significance in univariate Cox regression analysis (*P*<0.05). (D) Plots as in (C) within the BRCA cohort.


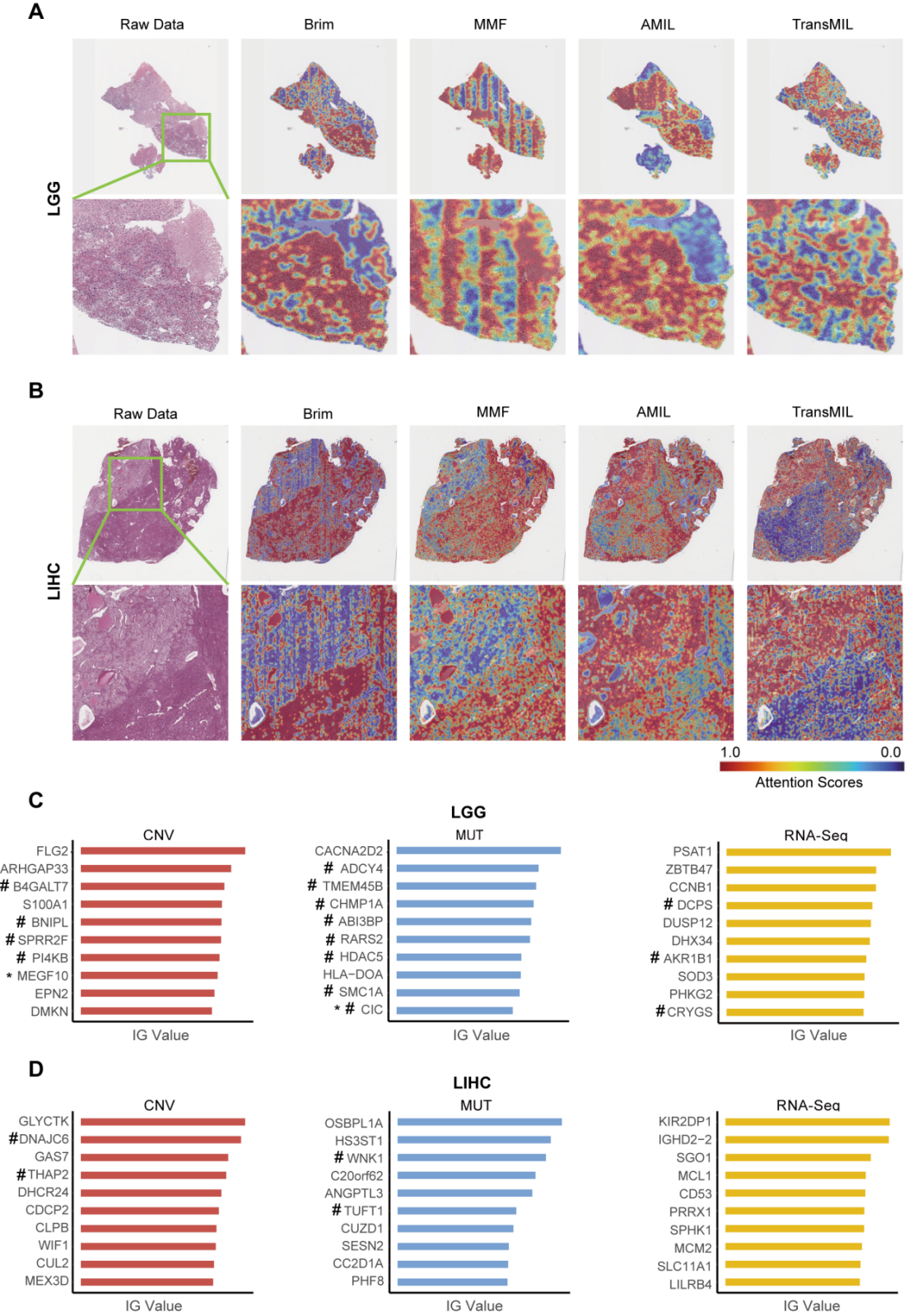


**Figure S9.** Model Interpretability and visualization in LGG and LIHC

(A) Represent WSI plots of AMIL, TransMIL, MMF and Brim models in LGG cohort, high-attention regions in the heatmaps of represent WSI plot correspond to high contributed morphological features in prognosis prediction. (B) Plots as in (A) within the LIHC cohort. (C) The calculated IG values of individual genomic molecular features based on the trained Brim model within the LGG cohort, * represents the identified genomic molecular features with literature validation, and # represents features with statistical significance in univariate Cox regression analysis (*P*<0.05). (D) Plots as in (C) within the LIHC cohort.


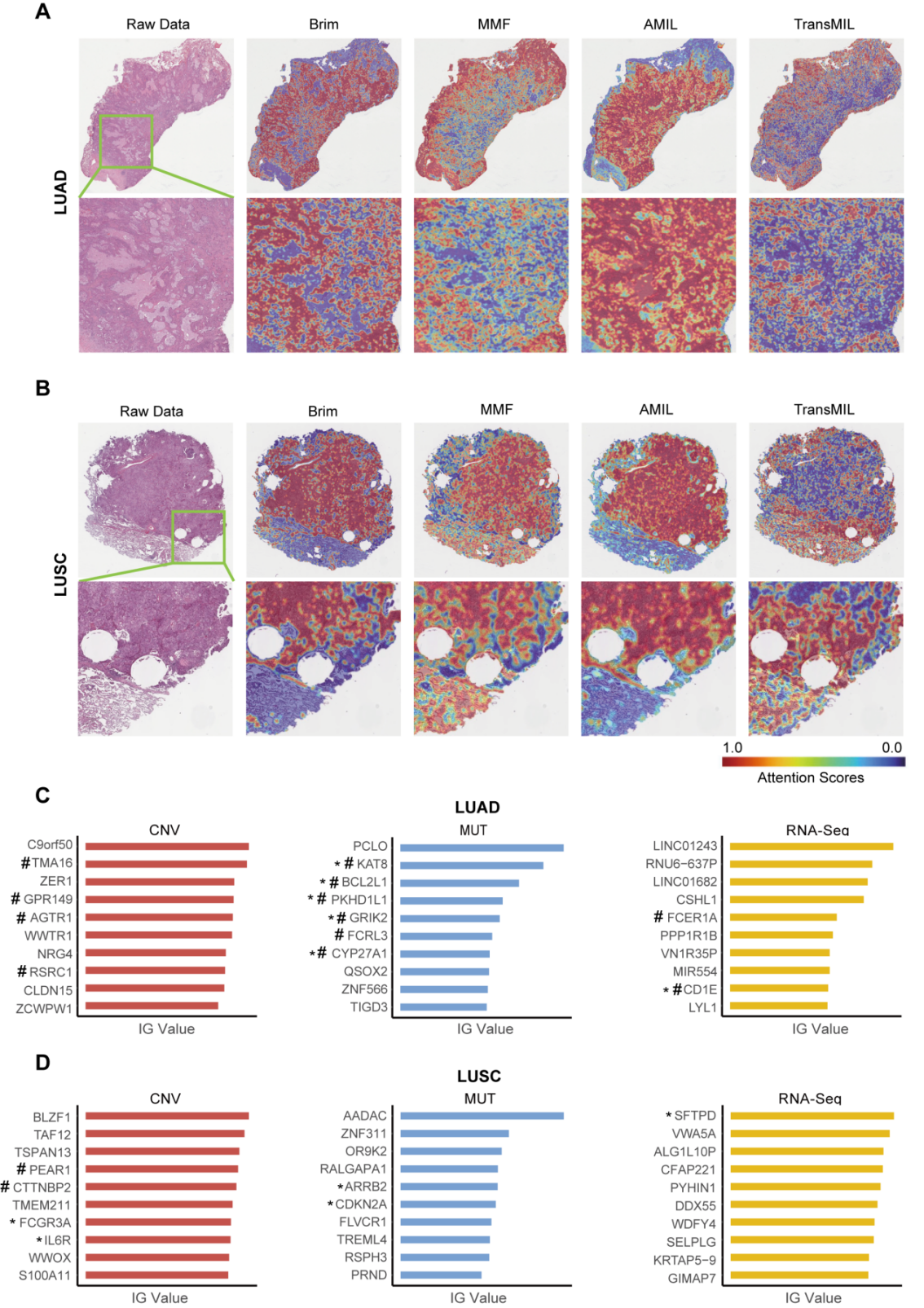


**Figure S10.** Model Interpretability and visualization in LUAD and LUSC

(A) Represent WSI plots of AMIL, TransMIL, MMF and Brim models in LUAD cohort, high-attention regions in the heatmaps of represent WSI plot correspond to high contributed morphological features in prognosis prediction. (B) Plots as in (A) within the LUSC cohort. (C) The calculated IG values of individual genomic molecular features based on the trained Brim model within the LUAD cohort, * represents the identified genomic molecular features with literature validation, and # represents features with statistical significance in univariate Cox regression analysis (*P*<0.05). (D) Plots as in (C) within the LUSC cohort.


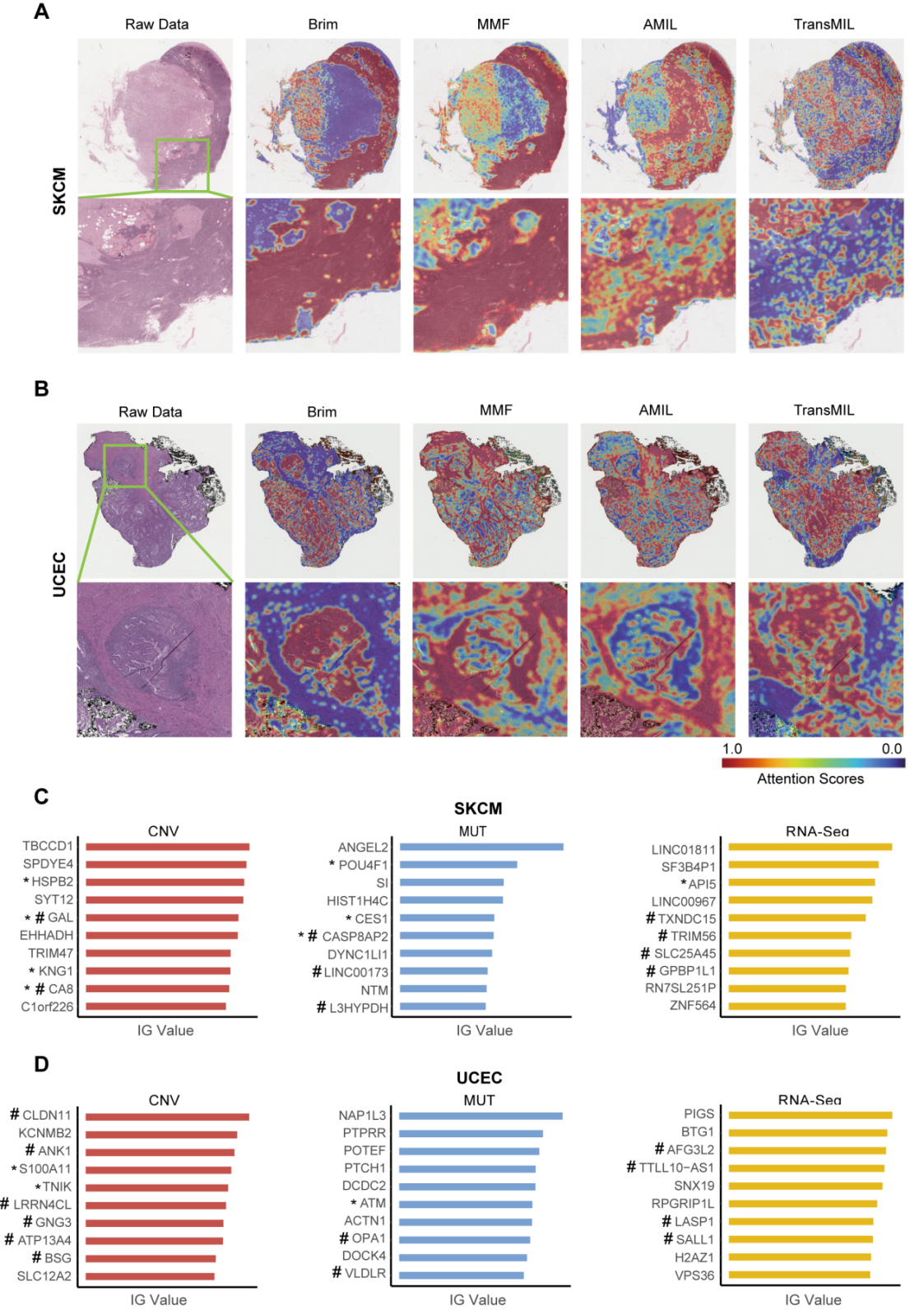


**Figure S11.** Model Interpretability and visualization in SKCM and UCEC

(A) Represent WSI plots of AMIL, TransMIL, MMF and Brim models in SKCM cohort, high-attention regions in the heatmaps of represent WSI plot correspond to high contributed morphological features in prognosis prediction. (B) Plots as in (A) within the UCEC cohort. (C) The calculated IG values of individual genomic molecular features based on the trained Brim model within the SKCM cohort, * represents the identified genomic molecular features with literature validation, and # represents features with statistical significance in univariate Cox regression analysis (*P*<0.05). (D) Plots as in (C) within the UCEC cohort.


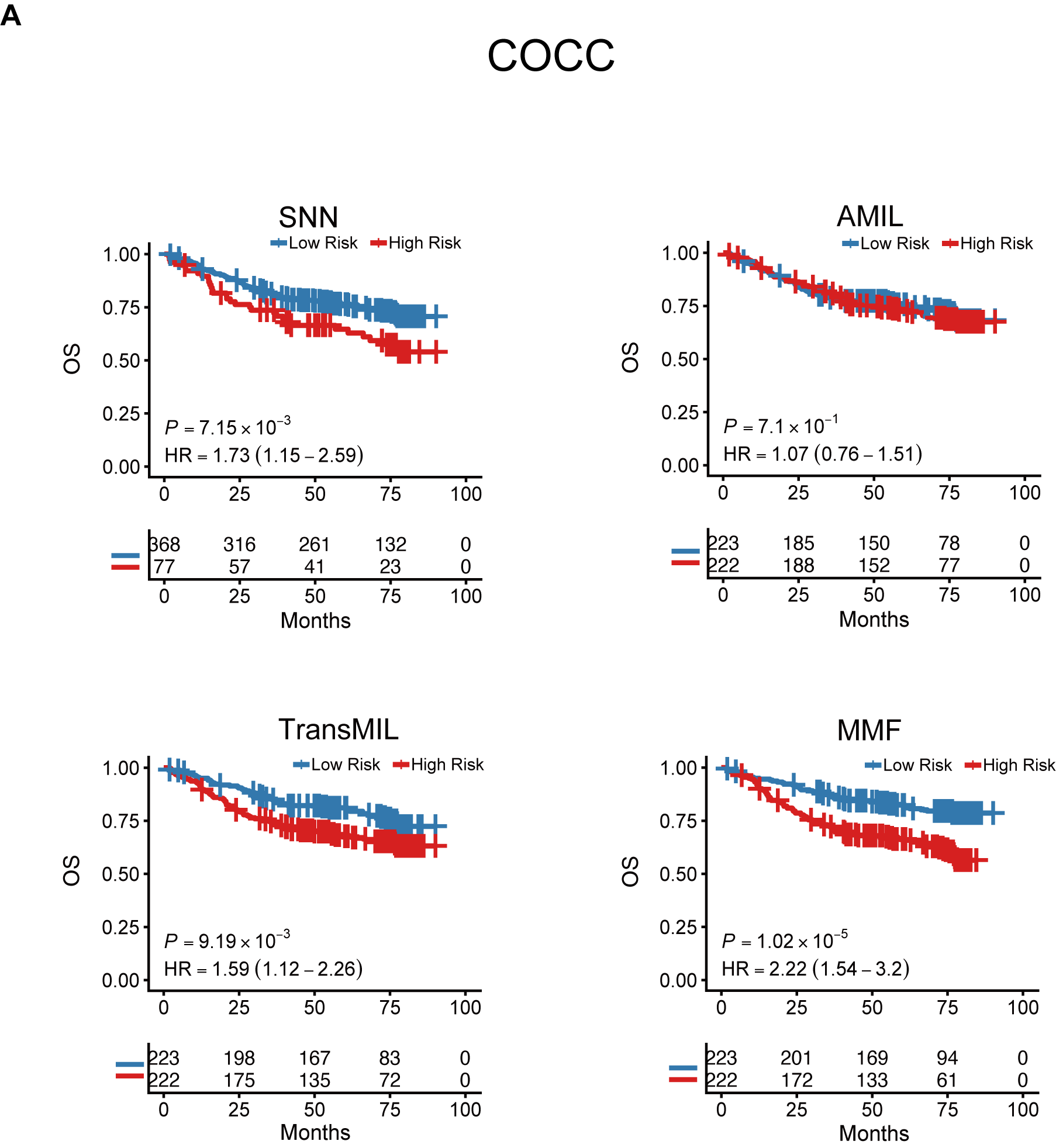


**Figure S12.** Model performance validation on COCC cohort

(A) Kaplan-Meier analysis of patient stratification into low and high-risk groups in the COCC cohort with complete multimodal data using SNN, AMIL, TransMIL, and MMF models. The median was used as the cutoff value. (n=445)

| **Table S1. C-index Model Performance Comparison between Brim and Other Bridge Models** | | | | | | | | | | | | | |
| --- | --- | --- | --- | --- | --- | --- | --- | --- | --- | --- | --- | --- | --- |
| TCGA-BLCA | | TCGA-BRCA | TCGA-COADREAD | TCGA-HNSC | TCGA-KIRC | TCGA-LGG | TCGA-LIHC | TCGA-LUAD | TCGA-LUSC | TCGA-SKCM | TCGA-STAD | TCGA-UCEC | Overall |
| **Single-Bridge** | | | | | | | | | | | | | |
|  | 0.636  (0.590-0.682) | 0.611  (0.550-0.672) | 0.640  (0.594-0.685) | 0.619  (0.579-0.659) | 0.687  (0.601-0.773) | 0.784 (0.694-0.875) | 0.662 (0.595-0.728) | 0.599 (0.563-0.635) | **0.618 (0.570-0.666)** | 0.586 (0.549-0.623) | 0.607 (0.574-0.641) | 0.659 (0.605-0.714) | 0.641 |
| **Non-Bridge** | | | | | | | | | | | | | |
|  | 0.648 (0.571-0.724) | 0.599 (0.478-0.721) | 0.618 (0.526-0.710) | 0.620 (0.579-0.661) | 0.691 (0.561-0.822) | 0.835 (0.772-0.898) | 0.653 (0.614-0.692) | 0.614 (0.553-0.676) | 0.597 (0.518-0.677) | 0.646 (0.614-0.678) | 0.597 (0.565-0.628) | 0.681 (0.642-0.720) | 0.648 |
| **Brim** | | | | | | | | | | | | | |
|  | **0.666**  **(0.620-0.712)** | **0.677**  **(0.604-0.749)** | **0.720**  **(0.681-0.759)** | **0.633**  **(0.607-0.659)** | **0.692**  **(0.612-0.773)** | **0.838**  **(0.819-0.858)** | **0.690**  **(0.635-0.745)** | **0.647**  **(0.605-0.689)** | 0.595  (0.545-0.645) | **0.660**  **(0.632-0.659)** | **0.640**  **(0.624-0.658)** | **0.723**  **0.706-0.739)** | **0.682** |

| **Table S2. C-index Model Performance Comparison between Brim and Other UNI Models** | | | | | | | | | | | | | |
| --- | --- | --- | --- | --- | --- | --- | --- | --- | --- | --- | --- | --- | --- |
| TCGA-BLCA | | TCGA-BRCA | TCGA-COADREAD | TCGA-HNSC | TCGA-KIRC | TCGA-LGG | TCGA-LIHC | TCGA-LUAD | TCGA-LUSC | TCGA-SKCM | TCGA-STAD | TCGA-UCEC | Overall |
| **UNI-ABMIL** | | | | | | | | | | | | | |
|  | 0.627 (0.596-0.657) | 0.685 (0.631-0.739) | 0.687 (0.665-0.709) | 0.620 (0.556-0.683) | 0.740 (0.662-0.818) | 0.787 (0.723-0.851) | 0.736 (0.669-0.803) | 0.590 (0.556-0.624) | **0.618 (0.600-0.637)** | 0.603 (0.550-0.655) | 0.616 (0.562-0.670) | **0.725 (0.682-0.769)** | 0.668 |
| **UNI-AVG** | | | | | | | | | | | | | |
|  | 0.612 (0.558-0.666) | **0.727 (0.659-0.795)** | 0.716 (0.662-0.769) | 0.591 (0.559-0.623) | **0.742 (0.663-0.821)** | 0.793 (0.701-0.885) | **0.742 (0.696-0.787)** | 0.593 (0.572-0.613) | 0.613 (0.567-0.658) | 0.593 (0.561-0.624) | 0.622 (0.539-0.705) | 0.696 (0.592-0.800) | 0.668 |
| **Brim** | | | | | | | | | | | | | |
|  | **0.666**  **(0.620-0.712)** | 0.677  (0.604-0.749) | **0.720**  **(0.681-0.759)** | **0.633**  **(0.607-0.659)** | 0.692  (0.612-0.773) | **0.838**  **(0.819-0.858)** | 0.690  (0.635-0.745) | **0.647**  **(0.605-0.689)** | 0.595  (0.545-0.645) | **0.660**  **(0.632-0.659)** | **0.640**  **(0.624-0.658)** | 0.723   0.706-0.739) | **0.682** |

| **Table S3.** Univariate Cox Proportional Hazard Regression Analysis for Brim Features | | | | |
| --- | --- | --- | --- | --- |
| COCC | | | | |
| Gene | beta | HR (95% CI for HR) | Wald.test | *P*-value |
| CLDN11 | 0.34 | 1.4 (1.1-1.8) | 7 | 0.0079 |
| GOLGA7B | 0.51 | 1.7 (1.2-2.3) | 11 | 0.0011 |
| MEDAG | 0.3 | 1.3 (1.1-1.6) | 10 | 0.0014 |
| SPART | 0.3 | 1.3 (1.1-1.7) | 6.6 | 0.0099 |
| MAFK | 0.5 | 1.6 (1.2-2.2) | 11 | 0.00091 |
| SPPL3 | 0.51 | 1.7 (1.1-2.4) | 7.2 | 0.0073 |
| SCAND1 | 0.29 | 1.3 (1.1-1.7) | 5.6 | 0.018 |

| COADREAD | | | | |
| --- | --- | --- | --- | --- |
| Gene | beta | HR (95% CI for HR) | Wald.test | *P*-value |
| IGFBP1 | 0.22 | 1.3 (1-1.5) | 4.4 | 0.035 |
| EPHX3 | 0.36 | 1.4 (1.2-1.8) | 11 | 0.0011 |

| BRCA | | | | |
| --- | --- | --- | --- | --- |
| Gene | beta | HR (95% CI for HR) | Wald.test | *P*-value |

| BLCA | | | | |
| --- | --- | --- | --- | --- |
| Gene | beta | HR (95% CI for HR) | Wald.test | *P*-value |
| IGHD2-2 | -0.16 | 0.85 (0.72-1) | 3.9 | 0.048 |
| PRRX1 | 0.12 | 1.1 (1-1.2) | 7.3 | 0.0069 |
| SPHK1 | 0.13 | 1.1 (1-1.3) | 8.2 | 0.0043 |
| SLC18A3 | 0.16 | 1.2 (1-1.3) | 5 | 0.025 |
| FAM71F2 | -0.33 | 0.72 (0.53-0.98) | 4.5 | 0.034 |

| HNSC | | | | |
| --- | --- | --- | --- | --- |
| Gene | beta | HR (95% CI for HR) | Wald.test | *P*-value |
| EPHA10 | -0.4 | 0.67 (0.49-0.92) | 6 | 0.015 |
| LOC100420324 | 1.1 | 2.9 (1.5-5.4) | 11 | 0.00097 |
| TWIST2 | -0.13 | 0.87 (0.77-1) | 3.9 | 0.048 |
| FXR1 | 0.29 | 1.3 (1.1-1.6) | 7.9 | 0.005 |
| ACOD1 | -0.53 | 0.59 (0.36-0.96) | 4.6 | 0.032 |

| LIHC | | | | |
| --- | --- | --- | --- | --- |
| Gene | beta | HR (95% CI for HR) | Wald.test | *P*-value |
| DNAJC6 | 0.29 | 1.3 (1.1-1.6) | 11 | 0.0012 |
| THAP2 | 0.73 | 2.1 (1.4-3.1) | 13 | 0.00028 |
| WNK1 | 0.3 | 1.4 (1.1-1.7) | 6.9 | 0.0084 |
| TUFT1 | 0.21 | 1.2 (1-1.5) | 4.5 | 0.034 |

| LUSC | | | | |
| --- | --- | --- | --- | --- |
| Gene | beta | HR (95% CI for HR) | Wald.test | *P*-value |
| PEAR1 | 0.19 | 1.2 (1-1.4) | 4 | 0.045 |
| CTTNBP2 | -0.14 | 0.87 (0.76-0.99) | 4.7 | 0.03 |

| STAD | | | | |
| --- | --- | --- | --- | --- |
| Gene | beta | HR (95% CI for HR) | Wald.test | *P*-value |
| XKR4 | 0.68 | 2 (1.1-3.5) | 5.5 | 0.019 |
| TRPC1 | 0.27 | 1.3 (1.1-1.6) | 6.9 | 0.0086 |
| TSHZ3 | 0.22 | 1.2 (1.1-1.5) | 6.4 | 0.011 |
| UNC79 | 0.75 | 2.1 (1.2-3.8) | 6.1 | 0.014 |
| LMO1 | 0.19 | 1.2 (1-1.4) | 4.9 | 0.026 |
| FFAR3 | 1.3 | 3.6 (1.5-8.8) | 8.1 | 0.0045 |
| RAB6C | 0.67 | 1.9 (1-3.7) | 4 | 0.045 |

| KIRC | | | | |
| --- | --- | --- | --- | --- |
| Gene | beta | HR (95% CI for HR) | Wald.test | *P*-value |
| CMC4 | 3.4 | 29 (4.1-210) | 11 | 0.00071 |
| GUCY1A1 | -0.21 | 0.81 (0.67-0.98) | 4.8 | 0.028 |
| OAZ2 | -0.42 | 0.66 (0.44-0.98) | 4.3 | 0.039 |
| GNG10 | -0.38 | 0.68 (0.52-0.9) | 7.4 | 0.0067 |
| FOXO1 | -0.47 | 0.62 (0.47-0.82) | 12 | 0.00068 |
| NBPF1 | -0.58 | 0.56 (0.39-0.8) | 10 | 0.0015 |
| UBTFL6 | -0.66 | 0.52 (0.37-0.73) | 15 | 0.00013 |
| KIF13A | -0.4 | 0.67 (0.51-0.87) | 8.8 | 0.003 |
| LSM7 | 0.62 | 1.9 (1.2-2.8) | 8.7 | 0.0031 |
| ITGA9 | -0.37 | 0.69 (0.56-0.84) | 13 | 0.00033 |
| FABP12 | 3.5 | 32 (2.1-490) | 6.2 | 0.013 |
| BRPF3 | -0.26 | 0.77 (0.61-0.98) | 4.4 | 0.035 |
| DCAF11 | -0.67 | 0.51 (0.35-0.75) | 12 | 0.00067 |

| LGG | | | | |
| --- | --- | --- | --- | --- |
| Gene | beta | HR (95% CI for HR) | Wald.test | *P*-value |
| DCPS | 0.45 | 1.6 (1-2.4) | 4 | 0.045 |
| AKR1B1 | 0.66 | 1.9 (1.2-3.1) | 8.1 | 0.0045 |
| CRYGS | 0.28 | 1.3 (1-1.7) | 5.4 | 0.02 |
| B4GALT7 | 0.62 | 1.9 (1.1-3.2) | 5.2 | 0.023 |
| BNIPL | 0.97 | 2.6 (1.5-4.8) | 11 | 0.0011 |
| SPRR2F | -4.1 | 0.017 (3e-04-0.96) | 3.9 | 0.048 |
| PI4KB | 0.78 | 2.2 (1.2-3.9) | 6.9 | 0.0088 |
| ADCY4 | 0.44 | 1.5 (1.1-2.2) | 6.7 | 0.0097 |
| TMEM45B | 0.38 | 1.5 (1-2.1) | 4.1 | 0.043 |
| CHMP1A | 0.73 | 2.1 (1.1-3.8) | 5.7 | 0.017 |
| ABI3BP | -0.15 | 0.86 (0.75-1) | 4.1 | 0.043 |
| RARS2 | 0.59 | 1.8 (1.1-2.8) | 6.6 | 0.01 |
| HDAC5 | -0.84 | 0.43 (0.28-0.67) | 14 | 0.00022 |
| SMC1A | 0.33 | 1.4 (1-1.9) | 5 | 0.025 |
| CIC | 0.65 | 1.9 (1.3-2.8) | 11 | 0.00093 |

| LUAD | | | | |
| --- | --- | --- | --- | --- |
| Gene | beta | HR (95% CI for HR) | Wald.test | *P*-value |
| FCER1A | -0.091 | 0.91 (0.84-1) | 4.2 | 0.041 |
| CD1E | -0.16 | 0.85 (0.76-0.96) | 7 | 0.0083 |
| TMA16 | 0.58 | 1.8 (1.3-2.4) | 13 | 0.00029 |
| GPR149 | 0.59 | 1.8 (1-3.2) | 4.1 | 0.043 |
| AGTR1 | -0.22 | 0.8 (0.64-0.99) | 4 | 0.045 |
| RSRC1 | 0.4 | 1.5 (1.1-1.9) | 8.9 | 0.0029 |
| KAT8 | -0.32 | 0.73 (0.53-0.99) | 4.2 | 0.042 |
| BCL2L1 | 0.38 | 1.5 (1.2-1.8) | 11 | 0.001 |
| PKHD1L1 | -0.91 | 0.4 (0.21-0.77) | 7.7 | 0.0056 |
| GRIK2 | 0.25 | 1.3 (1.1-1.6) | 6.5 | 0.011 |
| FCRL3 | -0.23 | 0.79 (0.66-0.96) | 5.7 | 0.017 |
| CYP27A1 | -0.14 | 0.87 (0.75-1) | 3.9 | 0.048 |

| SKCM | | | | |
| --- | --- | --- | --- | --- |
| Gene | beta | HR (95% CI for HR) | Wald.test | *P*-value |
| TXNDC15 | -0.29 | 0.75 (0.62-0.91) | 8.8 | 0.0029 |
| TRIM56 | -0.37 | 0.69 (0.57-0.83) | 15 | 0.00011 |
| SLC25A45 | -0.3 | 0.74 (0.6-0.92) | 7.1 | 0.0078 |
| GPBP1L1 | -0.23 | 0.79 (0.65-0.97) | 5 | 0.025 |
| GAL | 0.12 | 1.1 (1-1.2) | 10 | 0.0013 |
| CA8 | 0.13 | 1.1 (1.1-1.2) | 12 | 0.00045 |
| CASP8AP2 | -0.16 | 0.85 (0.73-0.99) | 4.2 | 0.042 |
| LINC00173 | -0.2 | 0.82 (0.68-0.99) | 4.1 | 0.043 |
| L3HYPDH | -0.34 | 0.71 (0.59-0.86) | 13 | 0.00039 |

| UCEC | | | | |
| --- | --- | --- | --- | --- |
| Gene | beta | HR (95% CI for HR) | Wald.test | *P*-value |
| AFG3L2 | 0.32 | 1.4 (1.1-1.8) | 6 | 0.014 |
| TTLL10.AS1 | -0.99 | 0.37 (0.19-0.72) | 8.6 | 0.0034 |
| LASP1 | 0.33 | 1.4 (1-1.8) | 5.2 | 0.022 |
| SALL1 | -0.18 | 0.83 (0.72-0.97) | 5.9 | 0.015 |
| CLDN11 | 0.28 | 1.3 (1.1-1.6) | 6.7 | 0.0096 |
| ANK1 | 0.21 | 1.2 (1-1.5) | 4 | 0.045 |
| LRRN4CL | -0.27 | 0.76 (0.61-0.95) | 6 | 0.014 |
| GNG3 | 0.59 | 1.8 (1.3-2.5) | 14 | 0.00019 |
| ATP13A4 | 0.39 | 1.5 (1.1-1.9) | 8.1 | 0.0044 |
| BSG | -0.43 | 0.65 (0.45-0.93) | 5.4 | 0.02 |
| OPA1 | 0.38 | 1.5 (1.1-1.9) | 6.4 | 0.011 |
| VLDLR | -0.3 | 0.74 (0.6-0.91) | 8.1 | 0.0044 |

| **Table S4.** Characteristics of patients in TCGA-COADREAD and COCC | | | |
| --- | --- | --- | --- |
|  | TCGA-COADREAD | COCC | *P*-Value |
| **Age** | | | |
| **<65years** | 189 (45.9%) | 280 (62.9%) | <0.001 |
| **≥65years** | 223 (54.1%)) | 165 (37.1%) |  |
| **Sex** | | | |
| **Female** | 200 (48.5%) | 191 (42.9%) | 0.114 |
| **Male** | 212 (51.5%) | 254 (57.1%) |  |
| **TNM_Stage** | | | |
| **I** | 76 (18.4%) | 56 (12.6%) | <0.001 |
| **II** | 139 (33.7%) | 183 (41.1%) |  |
| **III** | 127 (30.8%) | 109 (24.5%) |  |
| **IV** | 56 (13.6%) | 95 (21.3%) |  |
| **T Stage** | | | |
| **1** | 15 (3.6%) | 20 (4.5%) | 0.018 |
| **2** | 79 (19.2%) | 51 (11.5%) |  |
| **3** | 271 (65.8%) | 321 (72.1%) |  |
| **4** | 47 (11.4%) | 51 (11.5%) |  |
| **N Stage** | | | |
| **1** | 113 (27.4%) | 139 (31.2%) | 0.011 |
| **2** | 70 (17.0%) | 45 (10.1%) |  |
| **0** | 228 (55.3%) | 261 (58.7%) |  |
| **M Stage** | | | |
| **1** | 55 (13.3%) | 95 (21.3%) | 0.038 |
| **0** | 303 (73.5%) | 350 (78.7%) |  |
| **MSS/MSI Status** | | | |
| **MSS** | 284 (68.9%) | 378 (84.9%) | <0.001 |
| **MSI-H** | 126 (30.6%) | 48 (10.8%) |  |
| **CMS** | | | |
| **CMS1** | 44 (10.7%) | 51 (11.5%) | 0.308 |
| **CMS2** | 129 (31.3%) | 145 (32.6%) |  |
| **CMS3** | 42 (10.2%) | 48 (10.8%) |  |
| **CMS4** | 84 (20.4%) | 131 (29.4%) |  |
| **Location** | | | |
| **Right** | 189 (45.9%) | 96 (21.6%) | <0.001 |
| **Left** | 223 (54.1%) | 330 (74.2%) |  |

References

[1] Z. C. Shao, H. Bian, Y. Chen, Y. F. Wang, J. Zhang, X. Y. Ji, Y. B. Zhang, in *35th Conference on Neural Information Processing Systems (NeurIPS)* **2021**.

[2] Y. Xiong, Z. Zeng, R. Chakraborty, M. Tan, G. Fung, Y. Li, V. Singh, *Proc AAAI Conf Artif Intell* **2021**, *35* (16), 14138.

[3] S. G. Zadeh, M. Schmid, *IEEE Trans Pattern Anal Mach Intell* **2021**, *43* (9), 3126, <https://doi.org/10.1109/TPAMI.2020.2979450>.

[4] M. Sundararajan, A. Taly, Q. Yan, in *34th International Conference on Machine Learning* **2017**.

[5] T. U. Günter Klambauer, Andreas Mayr, Sepp Hochreiter, **2017**, <https://doi.org/https://doi.org/10.48550/arXiv.1706.02515>;

[6] M. Ilse, J. M. Tomczak, M. Welling, in *35th International Conference on Machine Learning (ICML)* **2018**;

[7] R. J. Chen, M. Y. Lu, D. F. K. Williamson, T. Y. Chen, J. Lipkova, Z. Noor, M. Shaban, M. Shady, M. Williams, B. J. Joo, F. Mahmood, *Cancer Cell* **2022**, *40* (8), 865, <https://doi.org/10.1016/j.ccell.2022.07.004>.
